# Marine Terpenoids from Polar Latitudes and Their Potential Applications in Biotechnology

**DOI:** 10.3390/md18080401

**Published:** 2020-07-29

**Authors:** Laura Núñez-Pons, Andrew Shilling, Cinzia Verde, Bill J. Baker, Daniela Giordano

**Affiliations:** 1 Department of Integrated Marine Ecology (EMI), Stazione Zoologica Anton Dohrn (SZN), Villa Comunale, 80121 Napoli, Italy; laura.nunezpons@szn.it; 2 Department of Chemistry, University of South Florida, Tampa, FL 33620, USA; ashillin@mail.usf.edu; 3 Institute of Biosciences and BioResources (IBBR), CNR, Via Pietro Castellino 111, 80131 Napoli, Italy; cinzia.verde@ibbr.cnr.it; 4 Department of Marine Biotechnology, Stazione Zoologica Anton Dohrn (SZN), Villa Comunale, 80121 Napoli, Italy

**Keywords:** Arctic/Antarctic, marine bioprospecting, marine natural product, terpene, terpenoid, biotechnological application, drug discovery

## Abstract

Polar marine biota have adapted to thrive under one of the ocean’s most inhospitable scenarios, where extremes of temperature, light photoperiod and ice disturbance, along with ecological interactions, have selected species with a unique suite of secondary metabolites. Organisms of Arctic and Antarctic oceans are prolific sources of natural products, exhibiting wide structural diversity and remarkable bioactivities for human applications. Chemical skeletons belonging to terpene families are the most commonly found compounds, whereas cytotoxic antimicrobial properties, the capacity to prevent infections, are the most widely reported activities from these environments. This review firstly summarizes the regulations on access and benefit sharing requirements for research in polar environments. Then it provides an overview of the natural product arsenal from Antarctic and Arctic marine organisms that displays promising uses for fighting human disease. Microbes, such as bacteria and fungi, and macroorganisms, such as sponges, macroalgae, ascidians, corals, bryozoans, echinoderms and mollusks, are the main focus of this review. The biological origin, the structure of terpenes and terpenoids, derivatives and their biotechnological potential are described. This survey aims to highlight the chemical diversity of marine polar life and the versatility of this group of biomolecules, in an effort to encourage further research in drug discovery.

## 1. Foreword

The global market for marine biotechnology is expected to reach US$ $6.4 billion by 2025 [1]. However, in regard to the polar regions, marine biotechnology has lagged and the potential for many sectors is not yet fully realized. These remote and underexploited habitats are promising sources of environmental and biomedical applications and may provide significant opportunities for compound discovery and bioprospecting [2,3,4,5,6].

The Arctic Ocean (~4.3% of Earth’s ocean area) and the meridional part of the Southern Ocean (south of the Antarctic Convergence, ~6.1% of Earth’s ocean area) comprise the Polar Seas [7]. In the coldest years, the sea ice cover in these areas can reach up to ~13% of Earth’s total surface. The Arctic Ocean is delineated by the International Hydrographic Organization (https://iho.int) as the body of water north of 57° from Earth’s equatorial plane. It is mainly characterized by light seasonality and cold temperatures with winter extremes, and comprises a vast ocean accessible to an influx of warm water from the Atlantic and the Pacific Oceans [8]. The Antarctic Ocean encompasses the body of water between 60° South latitude and the continent of Antarctica (https://iho.int). Known as being the driest, windiest and coldest place on Earth, the white continent is isolated from the other continents, geographically and thermally by the Antarctic Circumpolar Current. Its northern border, the Antarctic Polar Front, represents a barrier for latitudinal migration of marine organisms, which renders the local biota as highly endemic [9,10,11].

Some evidence indicates that the events that generated the confinement of Antarctica occurred about 34 million years ago around the Eocene/Oligocene limit [12]. Polar marine life has evolved to thrive at one of life’s extreme limits, with liquid water that cannot get any colder and long periods of darkness, which has selected for species with unique adaptations, including strategies based on often unique secondary metabolites [13,14].

Indeed, the distinctive environment and ecological pressures faced by marine wildlife in polar regions are major drivers of secondary metabolite production and the emergence of compounds with diverse biological activities [15]. Competition for space and food, predator avoidance, pathogen threat and, in general, intra-species chemical communication, promote chemical diversity in marine environments [16]. In addition, marine polar organisms require sophisticated biochemical and physiological adaptations to cope with low temperatures, strong winds, arrested nutrient availability and high UV radiation [6]. Therefore, under extreme polar scenarios, bioactive compounds in the form of secondary metabolites are of major relevance for the survival of marine organisms [13].

Although compounds with varied biotechnological properties have been identified in polar organisms, the most commonly reported activity is toward pathogenic infections. Discovering novel and efficient antimicrobial metabolites is imperative, due to the increasing emergence of antibiotic-resistant or even multidrug-resistant strains of microorganisms [17].

In this review, we first expose the framework regarding the regulations that govern access and benefit sharing requirements in polar environments, and secondly, we report bioactive compounds isolated from marine bacteria, fungi and macroorganisms inhabiting Arctic and Antarctic environments. In particular, the present survey focuses on marine terpenes and their derivatives harboring properties with potential impact on pharmacological remedies towards human diseases.

## 2. Marine Biotechnology: Governance, Access and Benefit Sharing in the Antarctic and Arctic Environments

Marine natural products have attracted growing commercial interest from the biotech sector, supporting a rich marine biodiscovery pipeline [18]. The global market for marine-derived pharmaceuticals is around US$5 billion [19]. However, despite successful discoveries, few drugs isolated from marine organisms have been approved by the US Food and Drug Administration (FDA) [19,20,21], e.g., anticancer drugs such as cytarabine (Cytosar-U^®®^) from the Caribbean sponge *Tectitethya crypta* (=*Cryptotethya crypta*) [22], trabectedin (Yondelis^®®^) from the ascidian *Ecteinascidia turbinata* [23], eribulin (Halaven^®®^) from the sponge *Halichondria okadai*, the antiviral vidarabine from the sponge *Tethya crypta* and the analgesic ziconotide (Prialt^®®^) from the sea snail *Conus magus* [22].

Technological advances are swiftly reducing sequencing costs as demonstrated by the exponential growth of data freely available of public repositories (INSDC, comprising GenBank, EMBL-EBI/ENA and DDBJ) [24]. Nevertheless, there is an urgent need for services able to handle and harmonize the information obtained by heterogeneous standards. In addition, stakeholders, academia, industry, funding agencies, infrastructure providers and scholarly publishers should promote the FAIR (Findable, Accessible, Interoperable and Reusable) concepts. As yet, the Nagoya Protocol on Access to Genetic Resources and the Fair and Equitable Sharing of Benefits Arising from their Utilization of 2010 (“Nagoya Protocol”) does not apply to some areas of the ocean [25].

The Convention on Biological Diversity (CBD) [19] defines bioprospecting as the exploration and development of knowledge of genetic and biochemical resources for commercial purposes.

From the perspective of protecting biodiversity, the CBD and the Nagoya Protocol are the most important agreements. The Nagoya Protocol aims to support and supplement domestic legislation by defining the genetic resources and the contractual obligations to be written in mutually agreed terms. The CBD introduced novel legal concepts such as biodiversity, ecosystems, genetic resources, biotechnology, benefit sharing and traditional knowledge. Applying the CBD in Antarctica is no simple matter and still requires more explicit regulation [26,27,28].

Antarctica represents a space exclusively dedicated to international cooperation where states are not able to exercise sovereign rights. Antarctica is governed by its own regional regime, the Antarctic Treaty System (ATS). Essential to the ATS is the Antarctic Treaty (AT, 1959), which applies to the area south of 60° South latitude. The ATS is composed of five main treaties: the AT, the Convention for the Conservation of Antarctic Seals (1971), the Convention on the Conservation of Antarctic Marine Living Resources (CCAMLR 1980), the Convention on the Regulation of Antarctic Mineral Resource Activities (1988) and the Madrid Protocol amendment to the AT (1991). Article III of the AT requires that parties cooperate by sharing information on research results. Article II of the AT acknowledges the importance of the freedom of scientific investigation [29,30].

Bioprospecting is a rapidly accelerating activity in Antarctica with new players such as Malaysia with strong commercial interests in Antarctic organisms [31,32]. Terrestrial and marine biological material from Antarctica can be accessed either by collecting specimens from the field or from ex situ collections of Antarctic Biological Material (ABM) held in institutions around the world [26]. Bioprospecting was first discussed within the ATS in 1999. Since then it has received regular attention at meetings of the Scientific Committee on Antarctic Research (SCAR) [33], Committee for Environmental Protection (CEP) and the Antarctic Treaty Consultative Meeting (ATCM). Despite being a consistent topic at every ATCM since 2002, there are still many gaps to manage bioprospecting expectations and activities in Antarctica. CCAMLR may regulate travel to Antarctica but cannot legislate commercial profit from bioprospecting.

Antarctica is a hot spot for bioprospecting. A recent study reported patents for 439 Antarctic species that illustrate the great diversity of potential applications from marine cold-adapted organisms [34].

Bioprospecting is also developing in the Arctic, linked to regional features and the role of enterprises both large and small. Five nations, Canada, Denmark, Norway, Russia and the United States, are currently seeking to increase their territory in the Arctic. Companies from North America, Norway, Iceland, Finland, Sweden, Denmark and the United Kingdom are actively developing new biotechnologies based on genetic resources found in the Arctic [35]. Biotechnology research in the Arctic is mainly focused on industrial processes, food technology, pollution control technologies, pharmaceutical and medical products and health related advancements. More than 40 companies are now involved in bioprospecting in the Arctic largely attracted by the unexplored space and unusual biology of Arctic organisms that promise to open new opportunities in applied research [36]. In 2008, Leary [37] noted the recent rise in the number of patents derived from Arctic genetic resources, though no new reviews have appeared in the intervening 10+ years. Norway is leading projects in marine Arctic bioprospecting mostly focused on the Arctic and sub-Arctic waters. In March 2007, the Research Council of Norway launched MabCent-SFI, a marine bioprospecting project to discover and develop high-value, marine-based products for antibacterial, anticancer, anti-inflammatory products, antioxidants and diabetes medicine, as well as novel cold-adapted enzymes.

## 3. Marine Compounds from Polar Regions

In 2018, 1554 new natural products from polar marine sources were reported, revealing a number of pharmacologically interesting activities [38]. Of the more than 30,000 publications on marine natural products that have been produced, less than 3% were focused on studies from polar environments, largely due to the difficult accessibility and logistics and associated costs [5,16]. The majority of these cold-water natural products up to 2005 came from marine macroorganisms (e.g., sponges, ascidians, mollusks and corals). After 2005, thanks to advances in sampling methodologies and increased access to previously inaccessible areas, natural products isolated from marine polar microorganisms increased from 22% to 71%. Indeed, microbes have turned into a prolific resource for novel chemistry and the sustainable production of bioactive metabolites, overcoming the problem of recollection of samples from the field, which is required for macroorganisms [5].

Polar microorganisms have been used in many biotechnological applications for the production of bioactive molecules, including enzymes [4] for industrial processes such as food technology and natural compounds with antimicrobial, anti-inflammatory and anticancer activities [5,16]. Moreover, cold-adapted fungi and bacteria are considered a source of (i) antifreeze proteins [39] to be used in the food industry [40,41,42], and for biomedical purposes [43,44]; (ii) extracellular polymeric substances (EPSs), already used in the textile, cosmetic and food industry [45,46], in the remediation of heavy-metal contaminated environments, including low temperature applications [47], and in the pharmaceutical industry [48]; (iii) polyunsaturated fatty acids (PUFA) as promising alternatives to fish oils in the food industry [39,49,50].

Natural compounds with antimicrobial, anti-inflammatory and anticancer activities have been isolated from marine invertebrates in the Arctic region [3,51,52,53] and in the marine Antarctic environment [54,55,56,57].

Bioactive compounds recovered from host invertebrates may be produced by their microbial symbionts in the marine environment [58,59]. Although the ecological function of bacteria-invertebrate interactions in polar areas is only poorly understood, it seems that microbial metabolites may have crucial roles in host-associated chemical defenses and in shaping the symbiotic community structure [60]. Fungi and bacteria isolated from polar sponges and algae, for instance, show wide spectrum antibacterial activity against many microorganisms [61,62,63,64,65,66,67,68,69].

Bacteria and fungi, as representative of microbial biodiversity, and macroorganisms, such as sponges, macroalgae, ascidians, corals, bryozoans, echinoderms and mollusks, are the target biological taxa of our review. In Table 1 we list purified marine natural products with elucidated structures isolated from organisms inhabiting the polar environments, focusing mostly on those evaluated for biological activity against human pathogens. We excluded studies evaluating crude extracts or impure fractions for bioactivity, while some relevant ecological activities have been highlighted in the table.

### 3.1. Marine chemical diversity in Polar Regions

In the Antarctic and Arctic marine environment, a huge reservoir of microbial biodiversity has been recognized as promising for the isolation of new antimicrobial metabolites [85,156,157,158,159]. Among marine bacteria, *Pseudoalteromonas haloplanktis* TAC125 is a potential untapped source of biologically active natural products [160]. This cold-adapted bacterium produces valuable bioactive secondary metabolites, such as anti-biofilm metabolites [160], antibiotics such as methylamine, a volatile compound active against *Burkholderia cepacia* complex strains [81] and anticancer compounds [82]. The biotechnological potential of *P. haloplanktis* TAC125 is related to its genus *Pseudoalteromonas*, ubiquitously distributed in almost all marine habitats. The recent reconstruction of the largest *Pseudoalteromonas* pangenome allowed the identification of *Pseudoalteromonas* genes for cold adaptation and the production of secondary metabolites, among others [161]. Other Antarctic strains belonging to the same genus have proven useful for cosmetic application, including Antarcticine^®®^ launched by LIPOTEC as an anti-aging product obtained from extracts of marine *Pseudoalteromonas antarctica* [162], and SeaCode^®®^, a mixture of EPS and other glucidic exopolymers produced by biotechnological fermentation of a *Pseudoalteromonas* sp. isolated in Antarctic waters [163].

On the macroscopic realm, several new natural products have been isolated from marine polar biota, some yielding encouraging properties for biotech discovery [6]. The Arctic bryozoan *Dendrobeania murrayana* revealed the presence of the guanidine alkaloid, dendrobeaniamine A, which was inactive in cytotoxicity, antimicrobial, anti-inflammatory and antioxidant assays [115]. The permethylated hexapeptide friomaramide is a potent inhibitor of liver-stage infection by *Plasmodium falciparum* [164]. Among all marine natural products coming from the polar seas, we will focus in this review on terpenoids and their derivatives (included in Table 1), because they represent one of the most important classes of bioactive molecules commonly found in polar organisms.

### 3.2. Marine Polar Terpenoids

In marine environments, terpenes, terpenoids and their derivatives display an array of diverse chemical structures, with promising biological activities [165,166,167,168]. Terpenes are hydrocarbons that represent a large family of natural compounds, which include primary and secondary metabolites metaphorically biosynthesized from five carbon isoprene units.

Terpenoids, which are sometimes called isoprenoids, are derivatives or modified terpenes. Although sometimes used interchangeably with “terpenes,” terpenoids are often polycyclic structures where methyl groups have been moved or removed, and oxygen-containing functional groups have been added. About 60% of known natural products are terpenoids [169].

Modification of the isoprene unit structures leads to a wide structural diversity of derivatives showing diverse bioactivities. Terpenes and terpenoids are often classified by the number of isoprene units added to the parent terpene, and divided into biogenetic subclasses, e.g., monoterpenes, sesquiterpenes, diterpenes, sesterterpenes, triterpenes and tetraterpenes, containing two, three, four, five, six and seven isoprene units, respectively. The triterpenes include sterols and steroids and their derivatives conjugated to other functional group while the tetraterpenes include carotenoids. There are also compounds that contain terpene fragments, such as prenylated polyketides (meroterpenes, indole alkaloids).

**Figure 1:**
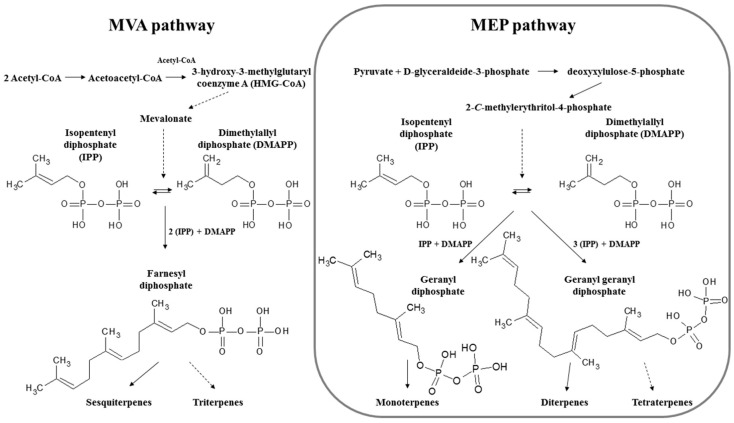
Schematic representation of isoprenoid biosynthesis by mevalonate pathway (MVA pathway) starting from two molecules of Acetyl-CoA and non-mevalonate pathway (MEP pathway) starting from pyruvate and glyceraldeide-3-phosphate. Adapted from [171].

Their biosynthesis was described in 1953, by Leopold Ružička as the C_5_ rule or biogenetic isoprene rule, reporting the linking of isoprene units “head to tail” to form chains [170]. There are two metabolic pathways that create terpenoids (Figure 1): (a) the mevalonic acid or mevalonate pathway (MVA pathway), which also produces cholesterol and occurs in the cytoplasm of most organisms (e.g., bacteria, archaea, fungi, plants, animals), except green algae; and (b) the 2-*C*-methyl-d-erythritol 4-phosphate/1-deoxy-d-xylulose 5-phosphate pathway (MEP/DOXP pathway), also known as non-mevalonate pathway, takes place in plastids of plants and green algae, in apicomplexan protozoa and in many bacteria [171]. From these two pathways isopentenyl diphosphate (IPP) and its isomer dimethylallyl diphosphate (DMAPP) are derived, which are sequentially elongated by prenyltransferases to geranyl diphosphate (C_10_), farnesyl diphosphate (C_15_) and geranylgeranyl diphosphate (C_20_) (Figure 1). These acyclic intermediates are then transformed by terpenoid synthases into monoterpenes, sesquiterpenes and diterpenes and further modifications produce triterpenes and tetraterpenes [171].

Marine organisms are prolific producers of terpenes and terpenoids. Particularly in cnidarians (corals), mollusks and sponges, these types of compounds are well represented among reported natural products. In ascidians and echinoderms, the proportion of terpene and terpenoid molecules reported to date is lower but they are still relatively common. In total, terpenoids are the most frequently reported natural product class from the Antarctic, displaying a large degree of structural diversity and bioactivity [172]. In the following paragraphs, we will describe some aspects of the biotechnological potential of terpene and terpenoid compounds from polar latitudes.

#### 3.2.1. Monoterpenes and Monoterpenoids

Monoterpenes are comprised of two isoprene units that result from a single condensation between DMAPP and IPP to yield a 10-carbon molecule, which is often further modified (Figure 1). Polyhalogenated monoterpenes are commonly produced by red algae inhabiting the shallow waters of the polar regions. These metabolites are often found in high abundance and can be either linear or cyclic. Like many natural products found in other phyla, the ecological function of these halogenated secondary metabolites is thought to be multi-faceted. The most commonly assumed role is that of defense, conveying deterrence against herbivory and resistance to detrimental fouling species [173]. These compounds may also be used offensively, possibly providing allelopathic competitive advantages to the producers, while deleteriously affecting potential competitors for space and resources [155,174,175,176].

**Figure 2:**
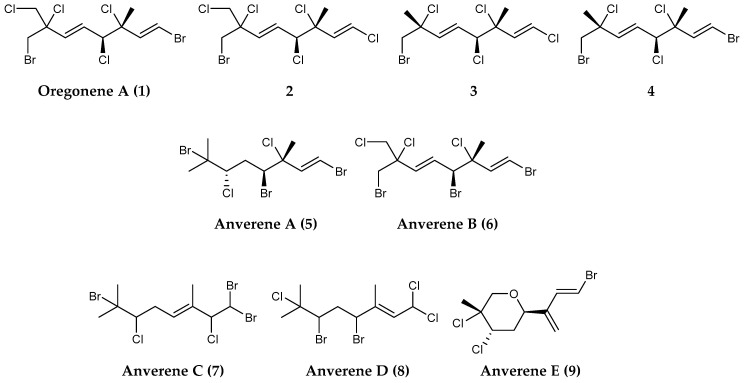
Structures of monoterpenoid compounds, (**1–9**) oregonene A and anverenes A–E from *Plocamium cartilagineum*.

Likely owing to their defensive and offensive ecological functions in nature, previous investigations into the therapeutic potential of halogenated metabolites in red seaweeds from other regions have yielded a plethora of relevant biological activities, ranging from antimicrobial to perhaps most notably their antitumor properties [155,174,175,176,177]. Similarly, a recent chemical investigation of what is generally considered to be an Antarctic subspecies of the red alga *Plocamium cartilagineum* collected from around Anvers Island along the Western Antarctic Peninsula yielded several polyhalogenated monoterpenes (**1–9**) including anverenes A-E (Figure 2), which displayed cytotoxicity in the low micromolar range (1–13 µM) against a cervical cancer cell line (HeLa) [154].

#### 3.2.2. Sesquiterpenes and Sesquiterpenoids

Sesquiterpenes comprise “one and a half monoterpenes” (*sesqui-* prefix means one and a half), thus, three isoprene units. They may be acyclic or contain rings, including many unique combinations. As in other terpenes, biochemical modifications such as oxidation or rearrangement produce the related sesquiterpenoids [169,171]. Sesquiterpenes and sesquiterpenoids are an important class of natural products with a wide range of biological activities as semiochemicals, e.g., defensive agents or pheromones, and commonly found in terrestrial organisms (e.g., plants, fungi and insects). Their occurrence in the marine environment is quite remarkable, with several ecological and pharmacological bioactivities described [178]. Therefore, marine sesquiterpenes and derivatives represent important candidates for natural products in drug discovery [166,179,180,181].

**Figure 3:**
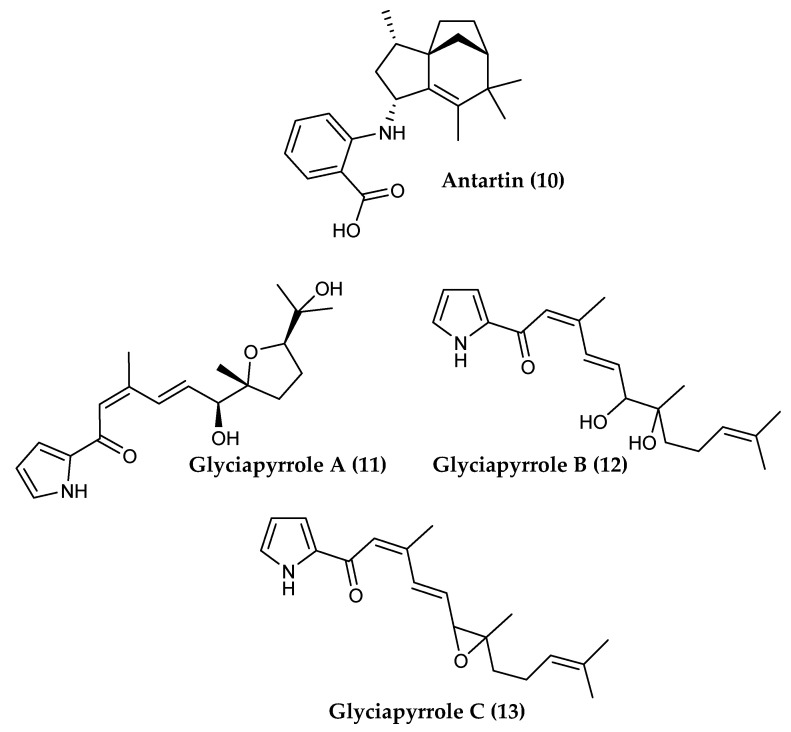
Structures of the sesquiterpenoid compounds (**10**) antartin A and (**11–13**) glyciapyrroles A–C from *Streptomyces* spp.

*Streptomyces* sp. SCO-736, isolated from an Antarctic marine sediment, was found to produce antartin (**10**) a tricyclic zizaane-type sesquiterpene with an unusual phenyl group (Figure 3) [79]. Tricyclic sesquiterpenes are an important class of marine natural products characterized by structural diversity often unprecedented when compared to terrestrial metabolites. They often display interesting biological activity, such as antifouling, cytotoxic, antibiotic, antifungal, antiparasitic and anti-inflammatory activities [182]. Antartin displays moderate cytotoxicity against a wide range of cancer cell lines, A549, H1299 and U87 cell lines, by causing cell cycle arrest at the G1 phase [79].

*Streptomyces* sp. NPS008187, isolated from a marine sediment in Alaska, produces three pyrrolosesquiterpenes, glyciapyrroles A (**11**), B (**12**) and C (**13**), along with the known diketopiperazines cyclo(leucyl-prolyl), cyclo(isoleucyl-prolyl) and cyclo(phenylalanyl-prolyl) (Figure 3). Glaciapyrrole A displays moderate antitumor activity against both colorectal adenocarcinoma HT-29 and melanoma B16-F10 tumor cell growth [80].

Although terpenoid substructures are often mixed with aromatic ring systems of various types (e.g., phenols, quinones, coumarines and flavonoids), they are rarely found in combination with pyrroles; there are only a few cases: pyrrolostatin, isolated from a Brazilian soil Actinobacteria *Streptomyces chrestomyceticus* EC40 [183], and two pyrrolosesquiterpenes isolated from soil actinomycete *Streptomyces* sp. Hd7–21 [184].

**Figure 4:**
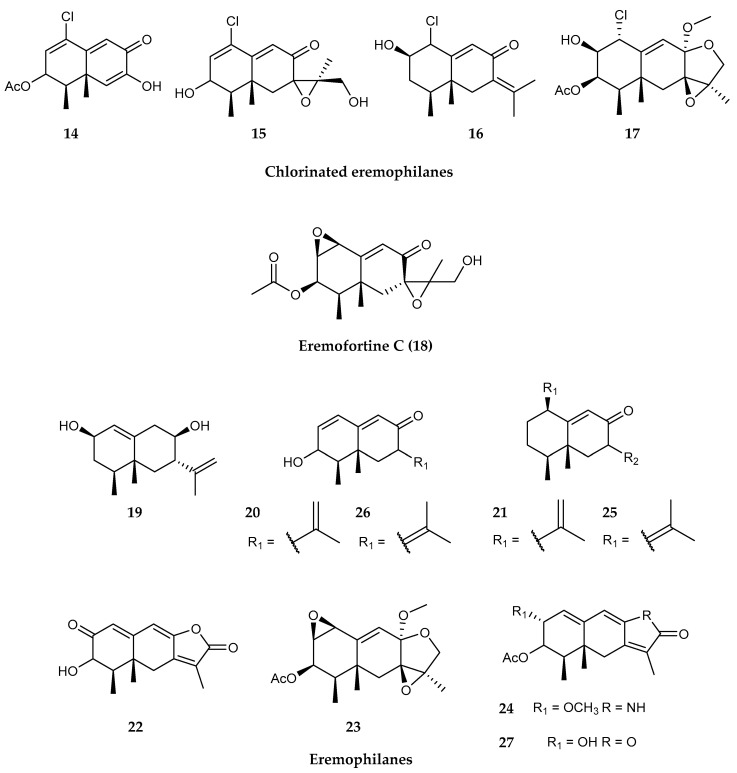
Structures of several sesquiterpenoid compounds from *Penicillium* sp. PR19N-1: (**14–17**) chlorinated eremophilane sesquiterpenes, (**18**) eremofortine C and (**19–27**) several eremophilane-type sesquiterpenes.

*Penicillium* sp. PR19N-1, a prolific producer of sesquiterpenes, is a fungus isolated from an Antarctic deep-sea sediment in Prydz Bay (−1000 m). It was found to contain four chlorinated eremophilane sesquiterpenes (**14–17**), along with a known sesquiterpenoid eremofortine C (**18**) [90] and five eremophilane-type sesquiterpenes (**19–23**), a new rare lactam-type eremophilane (**24**), together with three previously reported compounds (**25–27)** (Figure 4) [91]. Compound **14** displayed moderate cytotoxic activity against HL-60 and A549 cancer cell lines [90], whereas compound **23** was the most active against the A-549 cells [91]. Eremophilane-type sesquiterpenes, a subclass of sesquiterpenoids, firstly identified in plants [185] and only decades later in fungi [186], are characterized by complex and unique structure and diverse biological properties, such as phytotoxic, antifungal, antibiotic and anticancer bioactivity [179]. They are composed of a terpene skeleton, where the isoprene units are connected head-to-head [187]. They have also been found from a deep marine-derived fungus, *Aspergillus* sp. SCSIOW2, grown after chemical epigenetic manipulation to be induced to produce these secondary metabolites [188] in marine-derived *Xylariaceous* fungus LL-07H239 [189] and in the mangrove endophytic fungus *Xylaria* sp. BL321 [190]. However, these chloro-eremophilane sesquiterpenes are the first examples found in microorganisms [90], and compound **23** represents a rare example of eremophilane-type lactam found in microorganisms [91]. To date, only four lactam-type compounds were reported [191,192,193].

**Figure 5:**
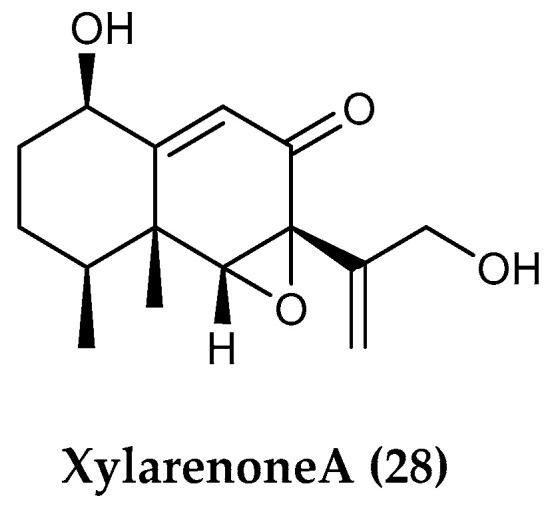
Structure of the sesquiterpenoid compound (**28**) xylarenone A from *Penicillium* sp. S-1-18.

*Penicillium* sp. S-1-18, a fungus isolated from Antarctic marine sediments, was reported to produce a new sesquiterpene guignarderemophilane F, with no detectable activity, together with six known compounds, among them the sesquiterpenoid xylarenone A (**28**) (Figure 5) [93]. Compound **28**, previously isolated from the endophytic fungal strain *Xylaria* sp. NCY2, exhibited moderate antitumor activities against HeLa and HepG2 (human liver carcinoma) cells and displayed growth-inhibitory effects against pathogenic microbes [94].

**Figure 6:**
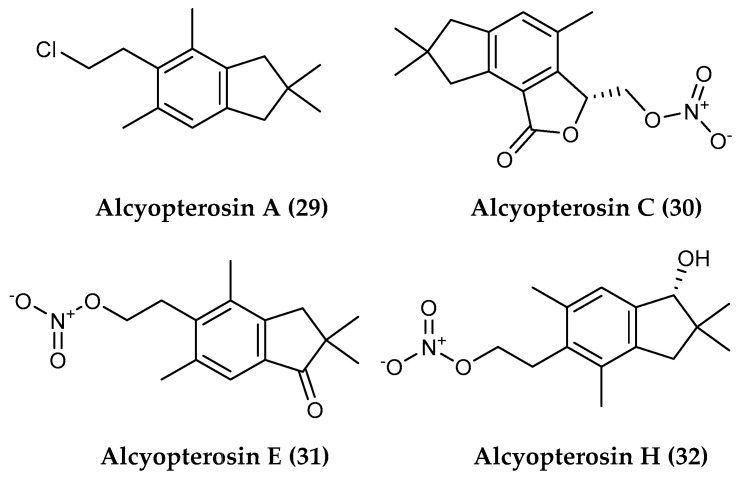
Structures of the sesquiterpenoid compounds (**29–32**) alcyopterosins A, C, E and H from *Alcyonium paessleri*.

Illudalane sesquiterpenes are a group of potent bioactive products, with antimicrobial and cytotoxic properties, that are primarily found in terrestrial ferns (family Pteridaceae) (e.g., [194]) and fungi (phylum Basidiomycota) (e.g., [195]). In the marine realm, 15 illudalane sesquiterpenoids, alcyopterosins (indicated with letter from A to O) were isolated from the sub-Antarctic soft coral *Alcyonium paessleri*, which was collected at a depth of 200 m near the South Georgia Islands. Alcyopterosins A (**29**), C (**30**), E (**31**) and H (**32**) (Figure 6) demonstrated mild cytotoxicity toward the Hep-2 (human larynx carcinoma) cell line with an IC_50_ of 13.5 µM, while alcyopterosins **29**, **30** and **32** displayed cytotoxicity toward HT-29 (human colon carcinoma) at an IC_50_ of 10 µg mL^−1^ [104].

**Figure 7:**
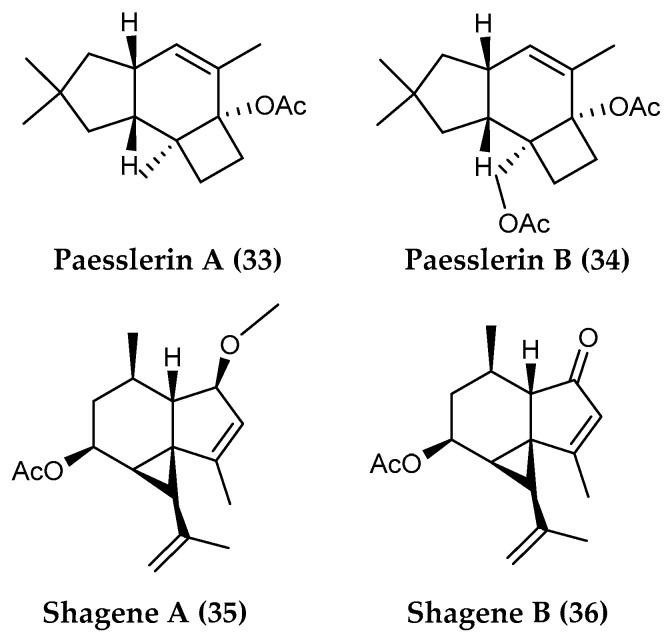
Structures of the sesquiterpenoid compounds (**33, 34**) paesslerins A–B from *Alcyonium paessleri* and (**35, 36**) shagenes A–B from an undescribed soft coral.

*Alcyonium paessleri* from South Georgia Island further yielded two new sesquiterpenoids, paesslerins A and B (**33** and **34**) (Figure 7), with moderate cytotoxicity against human tumor cell lines [105]. The structures shown are revised based on the results of total synthesis of the originally proposed structures [196].

Chemical examination of an undescribed soft coral collected from the Scotia Arc in the Southern Ocean resulted in the isolation and characterization of two new tricyclic sesquiterpenoids, shagenes A (**35**) and B (**36**) (Figure 7). Both compounds displayed significant inhibition against the visceral leishmaniasis, causing a parasite, *Leishmania donovani*, with no cytotoxicity against the mammalian host [113].

Octocorals or “horny corals” are conspicuous components on Antarctic sea bottoms, which extensively rely on natural products for protection. Eudesmane sesquiterpenes, ainigmaptilones A and B, seem to be taking part of the chemical arsenal of gorgonians *Ainigmaptilon antarcticus* from the Weddell Sea. Ainigmaptilone A was found to display ecologically relevant activities, in repelling putative sea star predators, and block fouling or microbial infections caused by sympatric bacteria strains and diatoms [197]. Antifouling properties have also been reported in azulenoid sesquiterpenes (i.e., linderazulene, ketolactone 2, and a new brominated C-16 linderazulene derivative) isolated from another Antarctic gorgonian, *Acanthogorgia laxa* [198]. The nudibranch gastropod, *Bathydoris hodgsoni*, from the Weddell Sea revealed high concentrations of a new drimane sesquiterpene, hodgsonal, exclusively allocated in the mantle. Hodgsonal has been hypothesized to be de novo synthesized and to play a defensive role, by analogy with drimane sesquiterpenes in other dorid nudibranchs [199,200].

#### 3.2.3. Diterpenes and Diterpenoids

Diterpenes are composed of “two monoterpene units,” or four isoprene subunits [171]. They are pharmacologically interesting compounds for possessing antimicrobial, antiviral, antiparasite, anticancer and anti-inflammatory properties [201].

**Figure 8:**
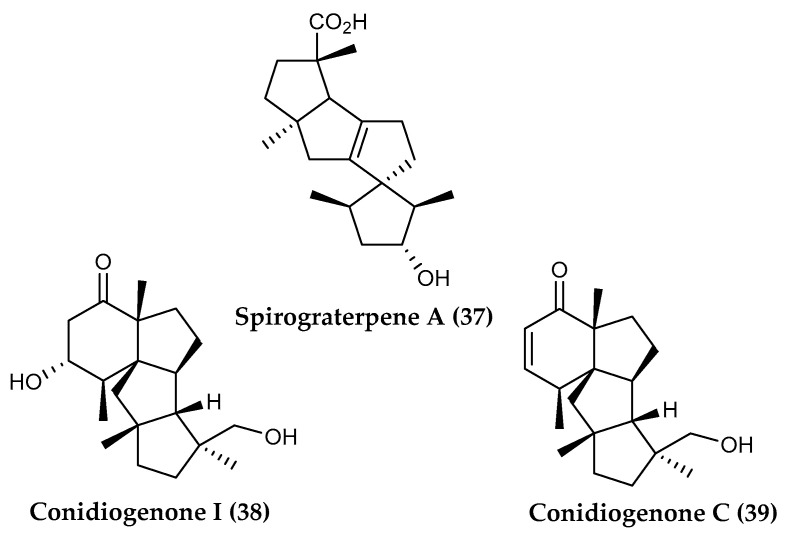
Structures of the diterpenoid compounds (**37**) spirograterpene A and (**38, 39**) conidiogenone I and C from *Penicillium granulatum*.

A rare spirocyclic diterpene, named spirograterpene A (**37**), was isolated from the deep-sea fungus *Penicillium granulatum* MCCC 3A00475 [92] from Prydz Bay, Antarctica, together with two known biosynthetically-related cyclopianes, conidiogenone I (**38**) [202] and conidiogenone C (**39**) (Figure 8) [203]. Spirograterpene A displays an anti-allergic effect on immunoglobulin E (IgE)-mediated rat mast RBL-2H3 cells, displaying 18% inhibition compared with the positive control, loratadine, with 35% inhibition at the same concentration of 20 μg/mL [92]. Cyclopianes, belonging to a rarely reported diterpenoid family, are tetracyclic diterpenes characterized by a highly fused and rigid ring system of 6/5/5/5 skeleton. They were first identified in the fungus *Penicillium cyclopium* in 2002 [204], followed by related compounds, all of which have been obtained only in the *Penicillium* species [202,203,204,205]. Spirograterpene A (**37**) is the second example of a diterpene spiro-tetracyclic skeleton with a 5/5/5/5 ring system [92], demonstrating that marine fungi represent a unique source of structurally novel compounds.

**Figure 9:**
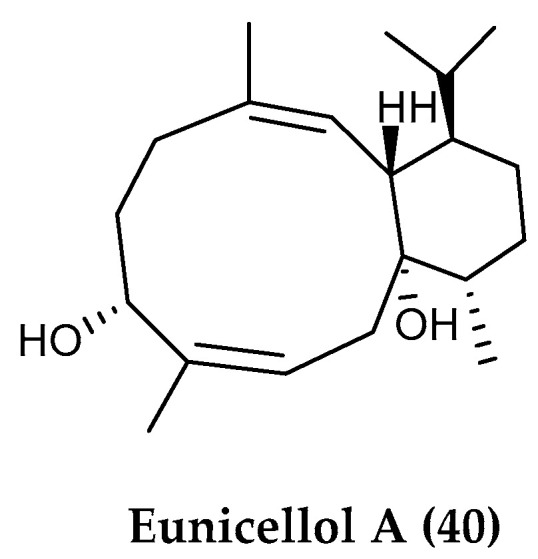
Structure of the sesquiterpene compound (**40**) eunicellol A from *Gersemia fruticosa*.

Among marine invertebrates, cnidarians, and in particular corals, have been found to possess a wide variety of diterpene and diterpenoid products, likely mediating allelochemical interactions [206]. Cold water polar ecosystems are devoid of scleractinian coral reef formations, such as those in the tropics, but instead harbor rich communities of soft-bodied octocorals that are well-known for their chemical diversity [16,207,208]. The Arctic soft coral *Gersemia fruticosa,* collected in the Bering sea, revealed the presence of three diterpenes named gersemiols A−C and another eunicellane diterpene, eunicellol A, which were purified together with the known sesquiterpene (+)-α-muurolene. Eunicellol A (**40**) (Figure 9) was found to exhibit moderate and selective antibacterial activity against methicillin-resistant *Staphylococcus aureus* (MRSA) [111].

**Figure 10:**
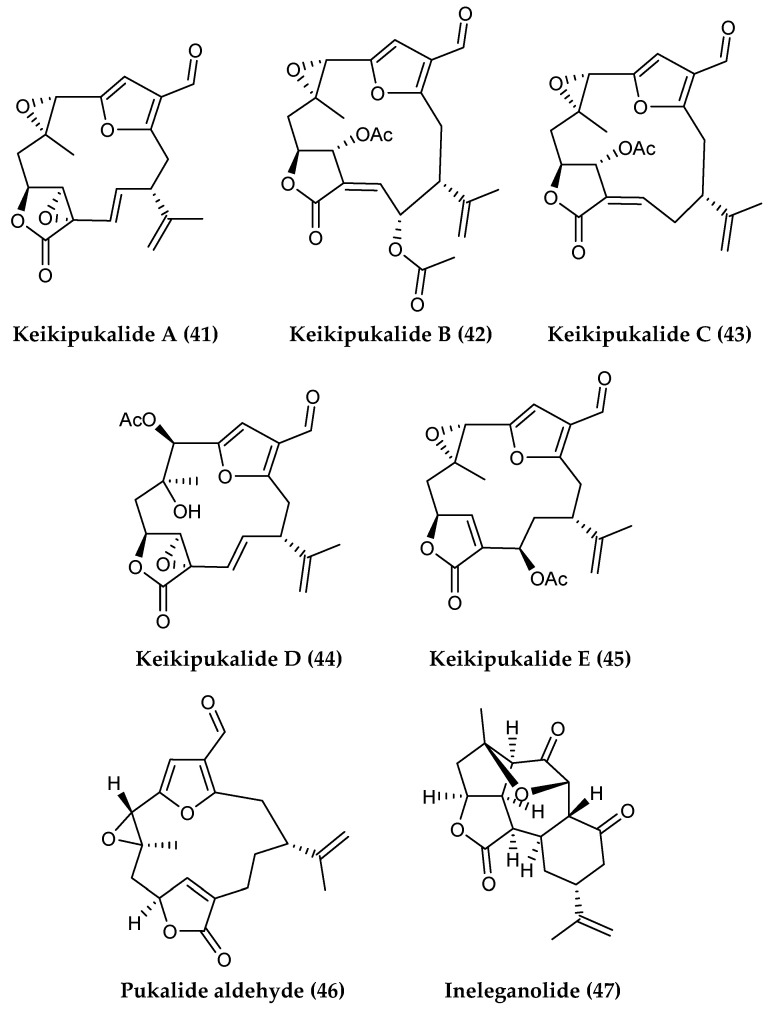
Structures of the diterpenoid compounds (**41–45**) keikipukalides A–E, (**46**) pukalide aldehyde and (**47**) ineleganolide from *Plumarella delicatissima*.

Five new furanocembranoid diterpenes, keikipukalides A−E (**41–45**), the known diterpene pukalide aldehyde (**46**) and the known norditerpenoid ineleganolide (**47**) (Figure 10) were isolated from the octocoral *Plumarella delicatissima* collected between 800 and 950 m depth, and demonstrated inhibitory activity against *L. donovani* [112].

**Figure 11:**
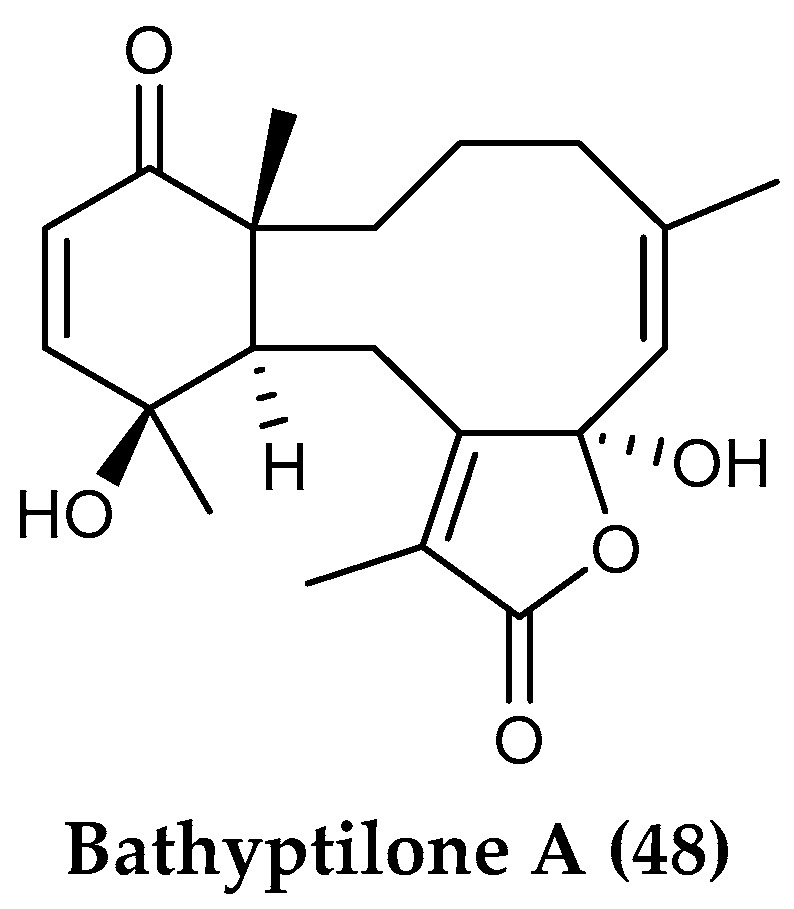
Structure of the diterpene compound (**48**) bathyptilone A from *Anthoptilum grandiflorum*.

The feather boa sea pen, *Anthoptilum grandiflorum*, is a cosmopolitan pennatulacean octocoral. Specimens collected at 662 and 944 m depth north of Burdwood Bank (Scotia Arc) yielded three new briarane diterpenes, bathyptilone A (**48**) (Figure 11), B and C together with a trinorditerpene, enbepeanone A. Nanomolar cytotoxicity against the neurogenic mammalian cell line Ntera-2 was detected only for bathyptilone A [107].

**Figure 12:**
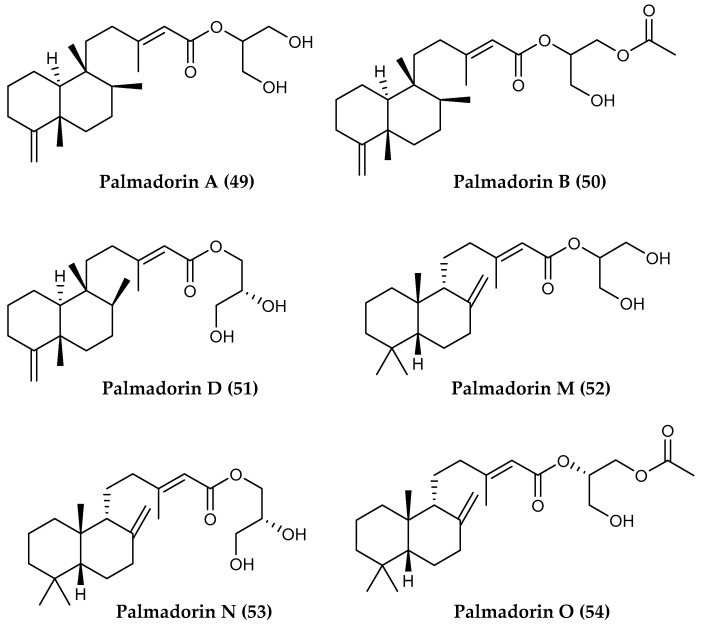
Structure of the diterpenoid compounds (**49–54**) palmadorins A, B, D, M, N and O from *Austrodoris kerguelenensis*.

The gastropod *Austrodoris kerguelenensis* is considered the most common and conspicuous Antarctic nudibranch, and among the most studied polar species for chemical ecology [13,14,209]. Detailed investigation of specimens from the vicinities of Palmer Station (Western Antarctic Peninsula), McMurdo Sound and the Weddell Sea has resulted in the isolation of a suite of tricyclic diterpenoid 2′-monoglyceryl esters (i.e., austrodorin A–B) [210] and diterpenoic acid glycerides [i.e 2′-acetoxyglyceryl (5R,10R,13R)-labda-8-en-15-oate, 3′-acetoxyglyceryl (5R,10R,13R)-labda-8-en-15-oate] [211], which were hypothesized to be produced by the nudibranch cells as opposed to being accumulated from its sponge diet [200]. Out of the variety of diterpene and diterpenoid products known from this mollusk, palmadorins A (**49**), B (**50**), D (**51**), M (**52**), N (**53**) and O (**54**) (Figure 12) proved to inhibit human erythroleukemia (HEL) cells and palmadorin M blocks Jak2, STAT5 and Erk1/2 activation in HEL cells, in addition to causing apoptosis [116].

**Figure 13:**
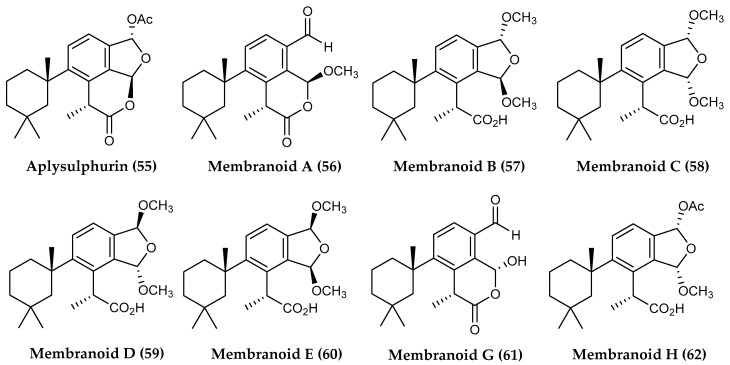
Structures of the diterpenoid compounds (**55**) aplysulphurin and (**56–62**) membranolids A, B, C, D, E, G and H from *Dendrilla membranosa*.

Sponges are known to be a particularly rich source of defensive diterpenoids, and among polar species, Antarctic *Dendrilla* sponges, typically reported as *D. membranosa* (recently revised to *D. antarctica*), stand out as the most prolific producers of bioactive diterpenes. While the chemical ecology of this sponge has been well-studied, investigations into the bioactive potential of Antarctic *Dendrilla* sponges was not carried out until 2004, with aplysulphurin (**55**) isolated from methanolic extracts along with three methyl acetals (**56, 58**, **60**) (Figure 13), which displayed moderate antifungal activity against *Candida albicans*, and antibiotic activity against *S. aureus* and *Escherichia coli* [123]. Termed “membranolides B–D” at the time of original publication, more recent investigations have shown these acetals to be artifacts from the methanolysis of aplysulphurin and have yielded several additional semisynthetic methyl acetal variations of the scaffold, now known collectively as membranoids A-H (**56–62**) (Figure 13). As a whole, the membranoids show potent bioactivity against the leishmaniasis causing parasite *L. donovani*, with membranoids B (**57**), D (**59**) and G (**61**) most notably displaying IC_50_ values of 0.8 µM, 1.4 µM and 1.9 µM, respectively, against *L. donovani* infected J774A.1 macrophages, with no discernable cytotoxicity observed towards the healthy variant of human cells [124].

**Figure 14:**
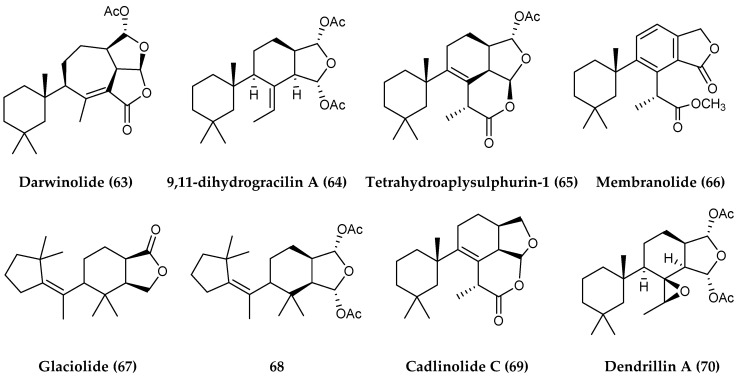
Structures of further diterpenoid compounds from *Dendrilla membranosa*: (**63**) darwinolide, (**64**) 9,11-dihydrogracilin A, (**65**) tetrahydroaplysulphurin-1, (**66**) membranolide, (**67**) glaciolide, (**68**), (**69**) cadlinolide C and (**70**) dendrillin A.

**Figure 15:**
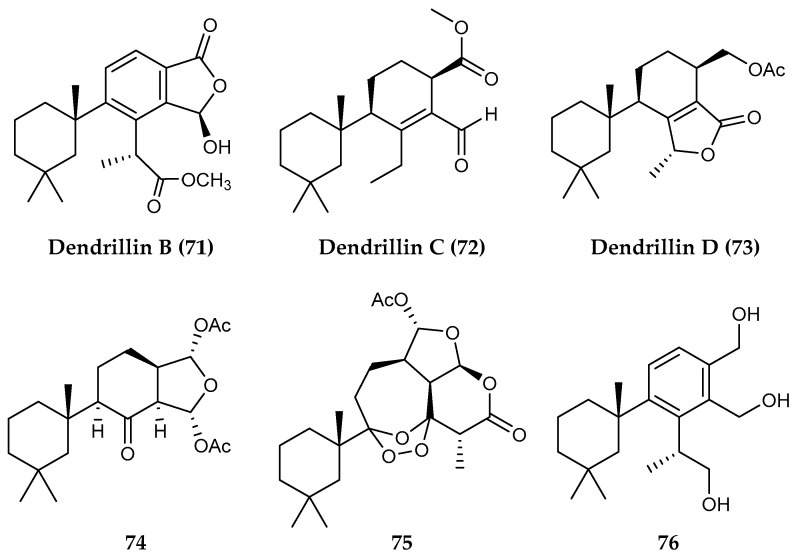
Structures of diterpenoid compounds from *Dendrilla membranosa*, (**71–73**) dendrillins B, C and D and semisynthetic derivatives (**74–76**).

*Dendrilla* sponges from around Anvers Island, Antarctica, have yielded several further diterpenoid natural products, including darwinolide (**63**), which was tested against MRSA and was found to be four times more potent against the biofilm (33.2 µM) than the planktonic form of MRSA (132.9 µM). This type of selective toxicity towards biofilms is rare and a promising lead in the search for antibiofilm-specific antibiotics [125]. A recent continuation of that study revealed a library of diterpenoids containing both known (**64–70**) (Figure 14) and new (**71–73**) natural products along with several additional semisynthetic derivatives (**74–76**) (Figure 15). This small but diverse collection of diterpenoids showed remarkable antibiotic properties against a range of infectious disease models. The most prominent bioactivity including membranolide (**66**) showed >90% eradication of MRSA biofilm at or below concentrations of 25 µg/mL, dendrillin B (**71**), active against *L. donovani* infected J774A.1 at macrophages at an IC_50_ of 3.5 µM, and **76** with 100% inhibition of *P. falciparum* at 5 µg/mL [126]. 9,11-dihydrogracilin A (**64**) isolated from *Dendrilla* sponges collected around the same area, has also recently been shown to display immuno-modulatory and anti-inflammatory properties in human cell lines [127].

#### 3.2.4. Sesterterpenes and Sesterterpenoids

**Figure 16:**
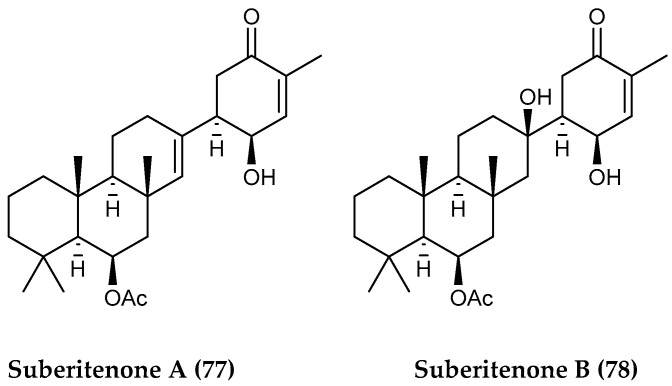
Structures of the sesterterpenoid compounds (**77, 78**) suberitenones A-B from *Suberites* spp.

Sesterterpenes are composed of “two and a half monoterpene units” and are typically C_25_, resulting from an initial condensation between DMAPP and isopentenyl IPP pyrophosphate followed by three additional and consecutive condensations of IPP to add to the growing chain. Sesterterpenes are the longest of the terpenes to be formed in this fashion, as subsequently longer terpenes with 30+ carbons are formed by additional condensation of two preformed phosphorylated isoprene precursors [212]. Sesterterpenoids are not as commonly found in the marine polar environment compared to diterpenes or triterpenes, however, sponges of the *Suberites* genus collected at several spots around Antarctica including King George Island and McMurdo Sound have yielded the polycyclic suberitenones A and B (**77**, **78**) (Figure 16), the latter of which has been shown to inhibit the cholesteryl ester transfer protein (CETP). This CETP protein mediates the transfer of cholesterol ester and triglyceride between high-density lipoproteins (HDL) low-density lipoproteins (LDL) and is a major target for the development of atherosclerotic disease treatments [142].

#### 3.2.5. Triterpenes and Triterpenoids

Triterpenes are formed by six isoprene units, conceptualized as three monoterpene units. Functionalized triterpenes (containing heteroatoms substitutions) should instead be referred to as triterpenoids. Animals, plants and fungi all produce triterpenes and triterpenoids, which exist in ~200 different skeletons and a great variety of structures (e.g., cholesterol) [213].

##### Steroids

Animals, plants and fungi all produce triterpenes, among which is squalene, the precursor to all steroids. These contain a core moiety of the triterpene cucurbitane, and in practice are biosynthesized from either lanosterol (animals and fungi) or cycloartenol (plants) via the cyclization of squalene. Steroids are further metabolized from squalene via subsequent demethylation to a tri-nor C_27_ skeleton, or further still to even smaller steroids. Steroids have two principal biological functions, being either key components of cell membranes or signaling molecules. Some examples of steroids are vitamin D3, the lipid cholesterol, the sex hormones estradiol and testosterone and the anti-inflammatory drug dexamethasone [214].

**Figure 17:**
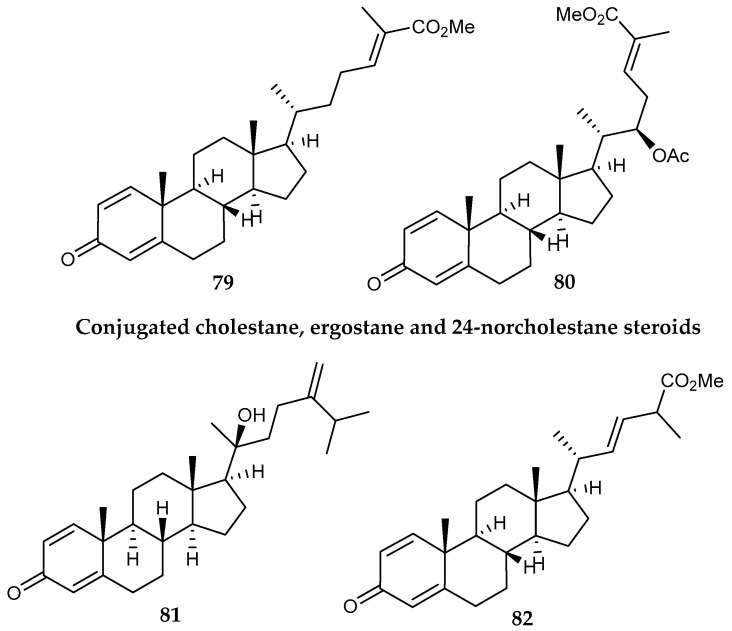
Structures of several steroid compounds *(***79–82**) from *Anthomastus bathyproctus*.

The Alcyonacean octocoral *Anthomastus bathyproctus* Bayer 1993 collected in the South Shetland Islands (Antarctica) afforded seven steroids, all of them displaying a cross-conjugated ketone system in the A ring of the tetracarbocyclic nucleus, while their side chains belong to the cholestane, ergostane and 24-norcholestane types. Compounds **79** to **82** (Figure 17) showed diverse in vitro cytotoxicity against the human tumor cell lines MDA-MB-231 (breast adenocarcinoma), A-549 (lung carcinoma) and HT-29 (colon adenocarcinoma) [106].

**Figure 18:**
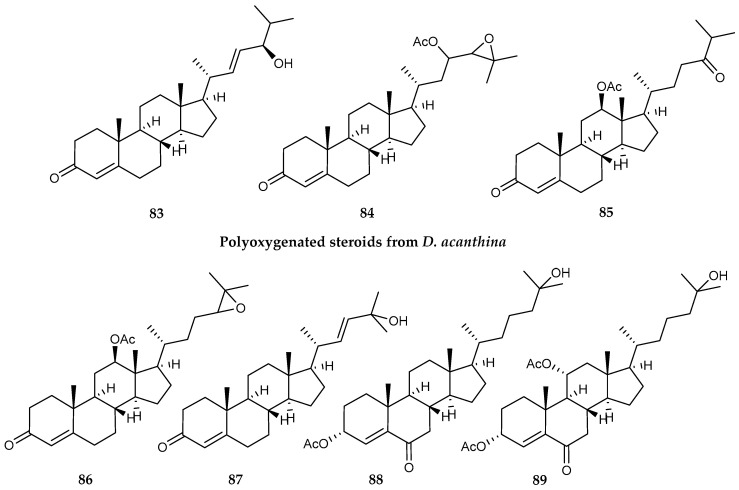
Structures of polyoxygenated steroid compounds *(***83–89**) from *Dasystenella acanthina*.

*Dasystenella acanthina* collected from Kapp Norvegia (Eastern Weddell Sea, Antarctica) was found to possess seven polyoxygenated steroids (**83–89**) (Figure 18). All compounds displayed some sort of growth inhibitory activity on tumor cell lines, including DU-145 (prostate carcinoma), LN-caP (prostate carcinoma), IGROV (ovarian adenocarcinoma), SK-BR3 (breast adenocarcinoma), SK-MEL-28 (melanoma), A549 (lung adenocarcinoma), K-562 (chronic myelogenous leukemia), PANC1 (pancreas carcinoma), HT29 (colon adenocarcinoma), LOVO (colon adenocarcinoma), LOVO-DOX (colon adenocarcinoma resistant to doxorubicin) and HeLa (cervix epithelial adenocarcinoma). The most affected cell lines were LN-caP and K-562. Compounds **88** and **89** presented broader cytostatic effects, and compound **89** was active against all tested cell lines [108].

**Figure 19:**
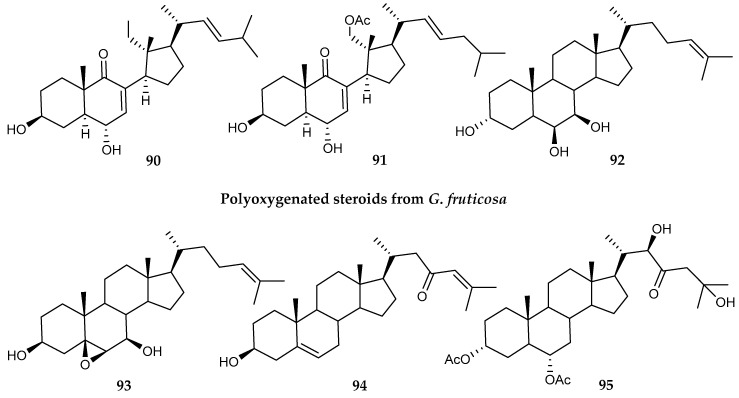
Structures of polyoxygenated steroid compounds *(***90–95**) from *Gersemia fruticosa*.

Six polyoxygenated sterols (**90–95)** (Figure 19) were isolated from the soft coral *Gersemia fruticosa*, exhibiting a moderate cytotoxic activity against human erythroleukemia K-562 cells and other leukemia cell lines [109].

**Figure 20:**
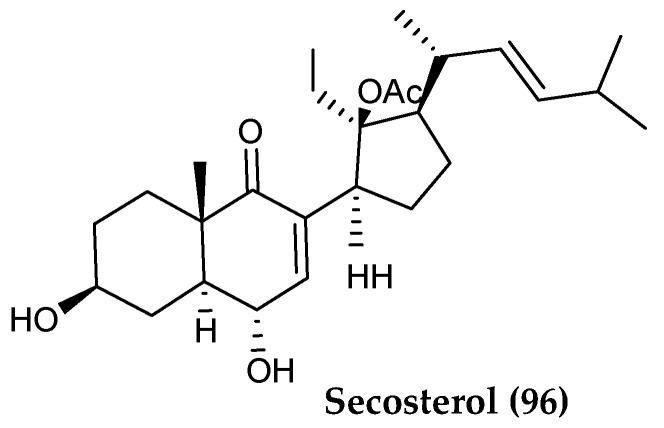
Structure of a further steroid compound *(***96**) from *Gersemia fruticosa*.

*Gersemia fruticosa* was also found to contain further a bioactive 9,11-secosterol steroid, named 24-nor-9,11-seco-11-acetoxy-3,6-dihydroxycholest-7,22(*E*)-dien-9-one (**96**) (Figure 20). This compound was shown to yield growth inhibition (IC_50_ below 10 µM) and cytotoxicity against human leukemia K562, human cervical cancer HeLa and Ehrlich ascites tumor cells in vitro [110].

**Figure 21:**
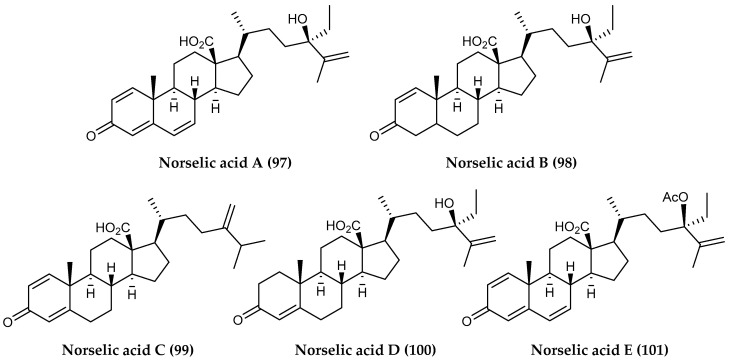
Structures of steroid compounds *(***97–101**) from *Crella* sp.

Sponges are known to produce a wide variety of bioactive steroids and steroid derivatives, and the species found in polar waters are no exception. A red encrusting sponge within the genus *Crella* is commonly found on the sheer walls in the shallow waters along the Western Antarctic Peninsula, and a group of specimens collected around Norsel Point near Anvers Island yielded norselic acids A-E (**97–101**) (Figure 21). Among these oxidized steroids, the most abundant, norselic acid A, showed inhibitory activity against MRSA and vancomycin-resistant *Enterococci faecium* (VREF) in addition to antifungal activity against *C. albicans*. All of the norselic acids showed low micromolar activity against the leishmaniasis causing protozoan parasite *L. donovani* with potencies ranging from 2.0–3.6 μM [122].

**Figure 22:**
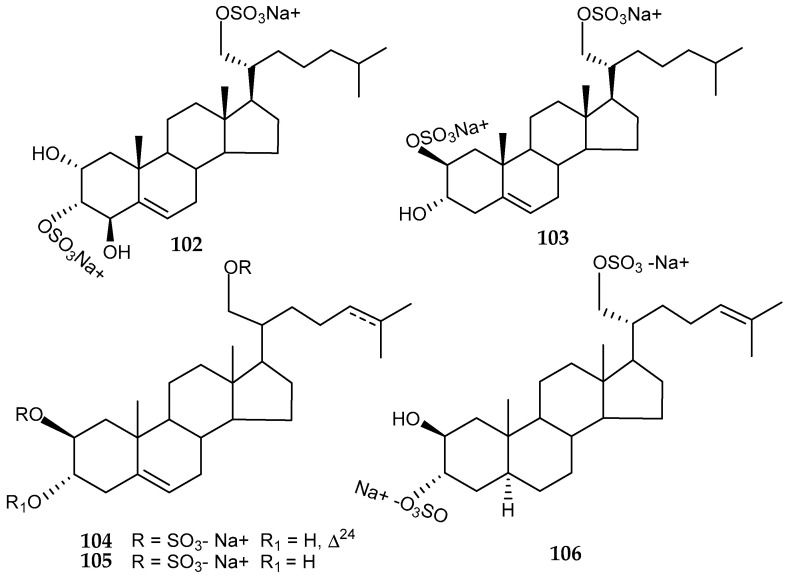
Structures of sulphated polyhydroxysteroids compounds *(***102, 103**) from *Ophiosparte gigas* and *(***104–106**) from *Astrotoma agassizii*.

Sulphated polyhydroxysteroids, obtained from Antarctic brittle star species, have been providing promising antiviral properties. Ophiuroid *Ophiosparte gigas*, coming from 70 m depth in the Ross Sea area revealed the presence of cholest-5-ene-2α,3α,4β,21-tetrao1-3,21-disulphate (**102**), which was remarkably cytotoxic, along with cholest-5-ene-2β,3α, 21-triol-2,21-disulphate (**103**) with cytoprotective activity against HIV-1 (Figure 22) [120]. Further disulfated polyhydroxysteroids (**104–106**) from another Antarctic ophiuroid, *Astrotoma agassizii* (Figure 22), as well as their synthetic derivatives displayed antiviral activities against one DNA (HSV-2) and two RNA (PV-3, JV) viruses [121].

**Figure 23:**
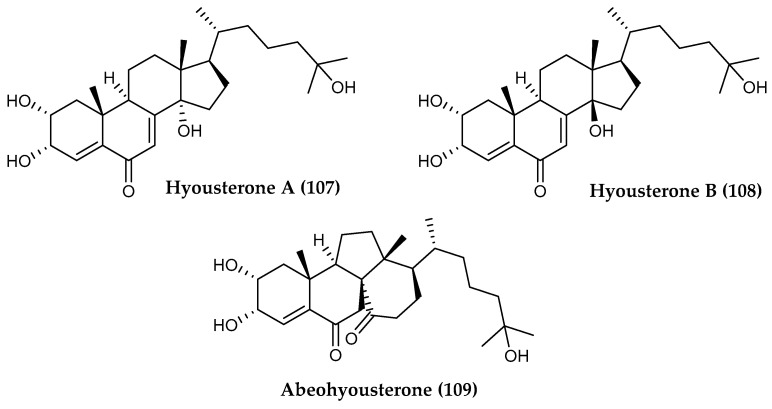
Structures of ecdysteroid compounds *(***107, 108**) hyousterones A-B and *(***109**) abeohyousterone from *Synoicum adareanum*.

Despite being most well-known for their role as hormones regulating molting in arthropods, a surprising array of unique ecdysteroids have been found in the Antarctic ascidian *Synoicum adareanum*, some of which display promising therapeutic activities. Among these are hyousterones A and C (**107**, **108**) (Figure 23) which displayed IC_50_ values of 10.7 µM and 3.7 µM, respectively, against the HCT-116 colon cancer cell line, while abeohyousterone (**109**) (Figure 23) was active at 3.0 µM in the same biological assay, all of which were isolated from tunicates around Anvers Island [149].

#### 3.2.6. Tetraterpenes and Tetraterpenoids

##### Carotenoids

Tetraterpenes are terpenes built from eight isoprene units (four monoterpene units). Carotenoids belong to the category of tetraterpenoids. They are natural isoprenoid pigments derived from head-to-tail condensation of two C_15_ or C_20_ isoprenoid precursors to form a C_30_ or C_40_ backbone, respectively, which are then modified to obtain different carotenoid structures [215]. They are divided into two main classes: carotenes, which are hydrocarbons, and xanthophylls, oxygenated derivatives of carotenes. Due to the long system of conjugated double bonds, they are able to capture and absorb light in the 400–500 nm range, displaying a peculiar strong coloration [216]. For this feature, carotenoids play a critical role in the photosynthesis process and provide photo-oxidative protection to the cells acting as strong antioxidant compounds. Being chemical quenchers of singlet oxygen, they function as potent scavengers of reactive oxygen species (ROS) [216]. They are essential constituents of photosynthetic organisms (e.g., plants, algae and cyanobacteria), but have been found also in fungi and bacteria [217]. Not synthesized by humans or animals, they are present in their blood and tissues, deriving from dietary sources. Being precursors of retinol (vitamin A), they perform a role of particular significance to human health [216].

In fact, carotenoids from marine environments are strong antioxidants used as nutraceutical ingredients in the food industry and cosmeceutical molecules for the photoprotection against UV radiation [218,219].

Although marine animals do not synthesize carotenoids de novo, they contain significant amounts of carotenoids derived from dietary sources. More than 100 carotenoids have been isolated from sponges, cnidarians, mollusks, crustaceans, echinoderms, tunicates and fishes [220].

Marine organisms inhabiting polar environments have developed a variety of adaptive strategies to cope with UV radiation, including light avoidance mechanisms, synthesis of UV-sunscreens, enzymatic and non-enzymatic quenching of ROS and DNA repair mechanisms [2]. The synthesis of carotenoid pigments in the Antarctic marine organisms belongs to the antioxidant defense mechanisms able to counteract ROS damage [2].

**Figure 24:**
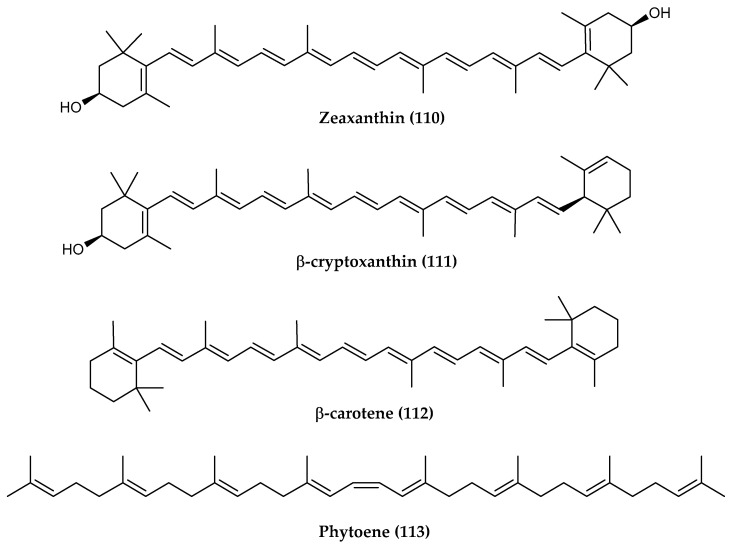
Structures of carotenoids found in bacteria *Cellulophaga fucicola* 416 and *Zobellia laminarie* 465: (**110**) zeaxanthin, (**111**) β-cryptoxanthin, (**112**) β-carotene and (**113**) phytoene.

Indeed, some pigmented bacteria owe their colors to the presence of carotenoids. The genome mining of *Marisediminicola antarctica* ZS314T, isolated from intertidal sediments of the cost near the Chinese Antarctic Zhongshan Station in East Antarctica, demonstrated the biosynthetic potential of this orange Actinobacterium in producing carotenoids and their derivatives [221]. *Cellulophaga fucicola* strain 416 and *Zobellia laminarie* 465, yellow and orange pigmented bacteria, respectively [98,99], isolated from Antarctic sea sponges, were found to be resistant to UV-B and UV-C radiation, thanks to the expression of carotenoids isolated and chemically identified by ultra-high-performance liquid chromatography with photodiode array and mass spectrometry detectors. Zeaxanthin (**110**), β-cryptoxanthin (**111**) and β-carotene (**112**) were identified in both strains, whereas two isomers of zeaxanthin was identified only in *C. fucicola* [98] and phytoene (**113**) only in *Z. laminarie* (Figure 24) [99]. These pigments displayed a very high antioxidant activity, although they were shown to be phototoxic in murine fibroblast lines [98,99].

**Figure 25:**
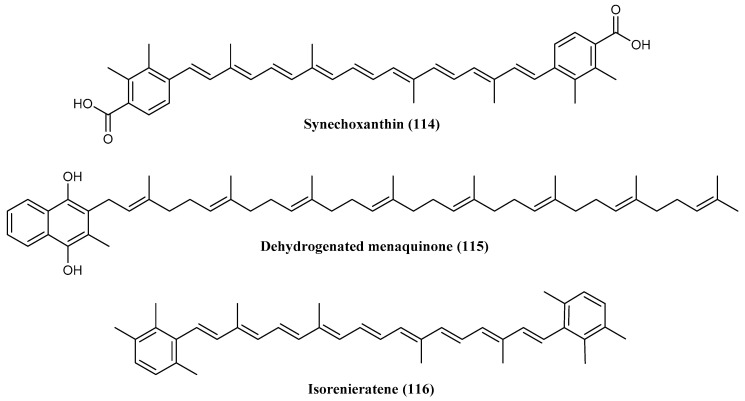
Structures of several carotenoids obtained from *Rhodococcus* sp. B7740: (**114**) synechoxanthin, (**115**) dehydrogenated menaquinone and (**116**) isorenieratene.

The red-orange strain *Rhodococcus* sp. B7740, isolated from 25 m deep-sea water in the Arctic Ocean, is a promising source of natural carotenoids and isoprenoid quinones, interesting both in amounts and varieties for the application in the food industry [74]. Among them, synechoxanthin (χ,χ-caroten-18,18′-dioic acid) (**114**), a unique aromatic dicarboxylate carotenoid, recently discovered only in some cyanobacteria [222,223], dehydrogenated menaquinones with eight isoprene units [MK-_8_(H_2_)] (**115**), produced in higher concentration than that reported in other bacteria [224], and isorenieratene (**116**), an aromatic carotenoid used in smear cheese industry [225], have been identified (Figure 25). The latter is a promising metabolite in future food and medicine applications for its higher stability than β-carotene and lutein in model gastric conditions and for its high retention rate in the gastrointestinal tract after ingestion [74]. Moreover, it has been demonstrated that isorenieratene, able to prevent UV-induced DNA damage in human skin fibroblasts [226], displays a photoprotective effect against UV-B radiation compared with two macular xanthophylls, lutein and zeaxanthin, in the multilamellar vesicles model and human retina cell model [75]. Additionally, MK-_8_(H_2_), the main menaquinone from *Rhodococcus* sp. B7740, has a potential application in the field of medicine for its higher antioxidant effect and antiglycation capacity compared with ubiquinone Q10 and MK_4_ [76].

#### 3.2.7. Triterpene and Triterpenoid Derivatives

##### Triterpenoid Conjugates

**Figure 26:**
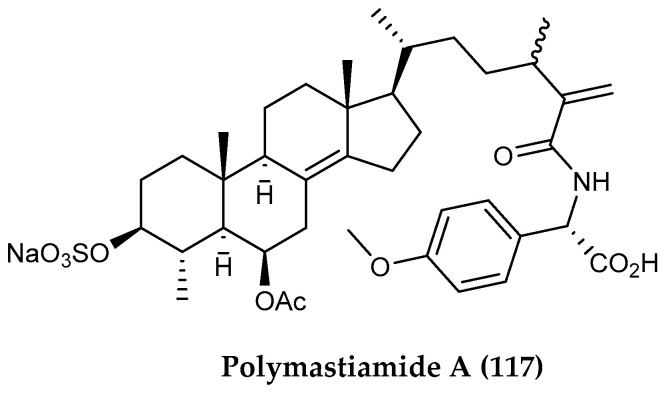
Structure of the triterpenoid conjugate compound (**117**) polymastiamide A from *Polymastia boletiformis*.

While steroids come in a variety of forms, they can also be found conjugated to other functional groups. One such example of these remarkable molecules are the sulfated steroid-amino acid conjugates known as polymastiamides A–F, isolated from the cold-water sponge *Polymastia boletiformis* collected at various locations along the Norwegian coast. Of these compounds, polymastiamide A (**117**) (Figure 26) has shown activity against plant pathogens *Cladosporium cucumerinum* and *Pythium ultimum* as well as human yeast pathogen *C. albicans* and antibacterial activity against *S. aureus* [138,139].

##### Triterpenoid Saponins

Triterpenoid saponins are amphipathic glycosides that have one or more hydrophilic glycoside moieties combined with a lipophilic triterpene or steroid derivative, thus, their chemical class can be identified as triterpenoid glycosides. They are well-known as plant-derived allelochemicals, but they have also been obtained from marine organisms, in particular sea cucumbers, where they have been proposed as chemotaxonomic proxies [227,228]. Several bioactive properties have been reported from these compounds, including anti-feedant (repellents), antimicrobial and ichthyotoxic [229,230]. The term saponin derives from the soapwort plant (genus *Saponaria* family Caryophyllaceae), the root from which is used as a soap. Additionally, these triterpene conjugates produce a soap-like foam when shaken in aqueous solutions [230]. The amphipathic properties of saponins make them efficient surfactants, due to their capacity to interact with cell membrane components, e.g., cholesterol and phospholipids, and therefore, they are interesting for the development of cosmetics, drugs and nutraceuticals (nutrient absorption enhancers) [231]. Saponins are also readily soluble in water, and have been proposed as adjuvants, to dissolve active principles in the development of vaccines [232].

**Figure 27:**
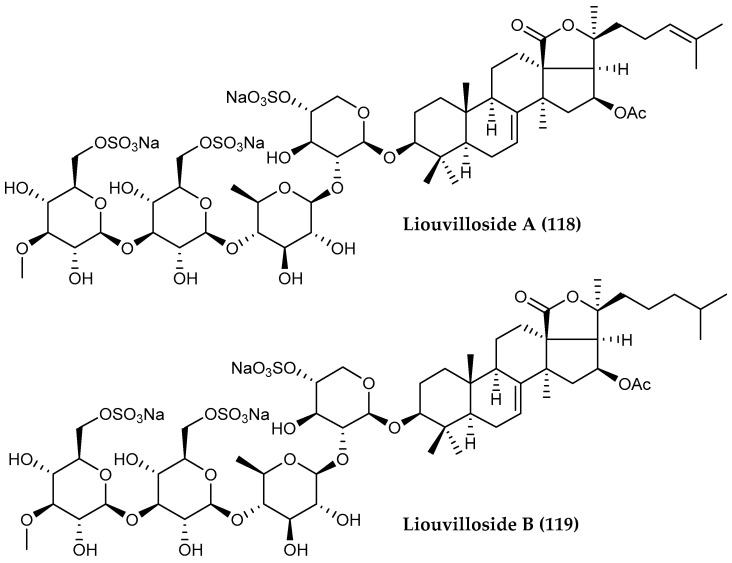
Structures of two triterpenoid saponin compounds (**118, 119**) liouvillosides A–B from *Staurocucumis liouvillei*.

Two trisulfated triterpene glycosides, liouvillosides A (**118**) and B (**119**) (Figure 27), both virucidal against herpes simplex virus type 1 (HSV-1), were isolated from the Antarctic cucumarid sea cucumber *Staurocucumis liouvillei,* collected in South Georgia Islands [118].

**Figure 28:**
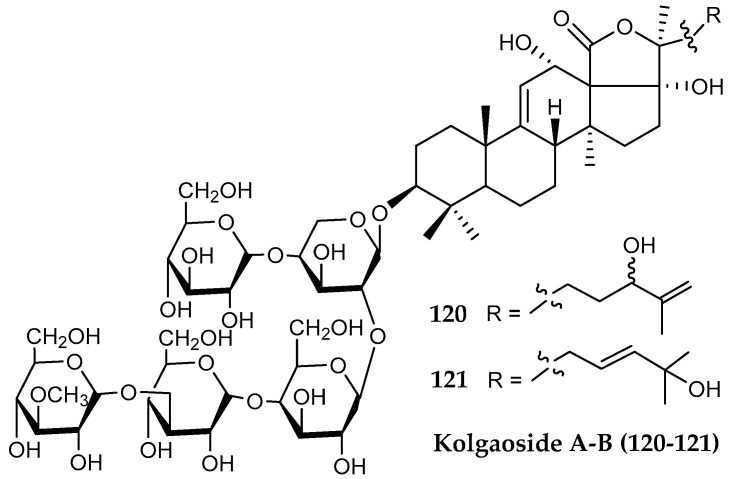
Structures of two triterpenoid glycoside compounds (**120, 121**) kolgaosides A–B from *Kolga hyalina*.

The deep sea Arctic holothurian echinoderm *Kolga hyalina* collected at Amundsen Basin at 4352–4354 m depth was found to contain holothurinoside B (known from temperate holothurid species [233], as well as two novel triterpene holostane nonsulfated pentaosides, kolgaosides A (**120**) and B (**121**) (Figure 28), both possessing hemolytic activity against mouse erythrocytes, and mild inhibitory action against Ehrlich ascite carcinoma cells. All these triterpene glycosides are structurally close to achlioniceosides A1–A3 from the Antarctic sea cucumber *Rhipidothuria racovitzai* Hèrouard, 1901 (=*Achlionice violaescupidata* [234]), supporting a potential chemotaxonomic value [235].

##### Meroterpenes

**Figure 29:**
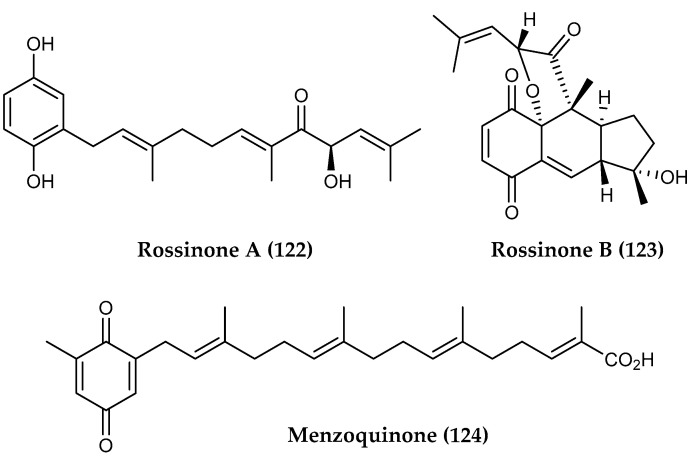
Structures of two meroterepenoid compounds, (**122, 123**) rossinones A-B from *Aplidium* spp., and (**124**) menzoquinone from *Desmarestia menziesii*.

Meroterpenes are molecules with a partial terpenoid structure attached to a shikimate-derived aromatic, usually a phenol, and are commonly found in marine ascidians, sponges and to a lesser extent in soft corals [236]. Bioactive meroterpenes featuring sesquiterpene moieties can also be found in organisms inhabiting the harsh polar regions. Tunicates in the genus *Aplidium* collected in the Ross Sea yielded rossinones A and B (**122, 123**) (Figure 29), which displayed antiproliferative activity against several cell lines with IC_50_ values ranging from 0.084 to 30 μM as well as selective antiviral activity against the DNA virus HSV-1 in addition to antibacterial and antifungal activity towards *Bacillus subtilis* and *Trichophyton mentagrophytes* [144].

While the majority of compounds reported from polar marine algae are monoterpenes, bioactive diterpenoids in the form of meroterpenes such as menzoquinone (**124**) (Figure 29) have been isolated from *Desmarestia menziesii*, a commonly occurring brown algae that plays a major role in structuring the benthic ecosystems along the northern latitudes of the Western Antarctic Peninsula. This methylated diterpene-quinone bearing a carboxylic acid has shown antimicrobial activity against MRSA and VREF [155].

## 4. Perspectives and Conclusions

Bioprospecting is a complex issue as it embraces many fields, such as intellectual property rights, scientific research and exploitation of resources in an eco-friendly ethical manner [237,238,239,240]. Cooperation is needed to develop sustainable, respectful and appropriate access and benefit-sharing mechanisms for marine resources as well as the promotion of the participation by all states in international negotiations for encouraging innovation and greater equity [241,242,243].

Bioactive natural products from the sea are in this particular era are timely to develop drugs to fight against ever more frequent and contagious emerging pathogenic agents. One actual example is a potent antiviral molecule obtained from the ascidian *Aplidium albicans*, which is under clinical trials on infected humans with Corona Virus, similar to the 2020 pandemic SARS CoV-19 [244].

Marine organisms from the polar regions could greatly contribute to this growing repertoire of promising bioactive compounds. Indeed, extreme environments are important hot spots of microbial, metazoan and symbiotic cluster diversities, where selective forces have promoted the evolution of unique biosynthetic pathways for secondary metabolite production [14]. In the light of the publication record on molecules with pharmacological potential isolated up to date from Arctic and Antarctic marine taxa, terpene and terpenoid derivatives seem to be the most frequently reported [5]. Furthermore, these compound types often yield remarkable antimicrobial properties, including anti-viral and antitumoral activities. Such cytotoxic actions likely respond to detrimental effects driven by terpenoid products on the structure and function of microbial membranes and cell walls [245]. Therefore, natural bioactive terpenes and terpenoids, in these times of increasing incidence of emerging infectious diseases, antibiotic-resistance pathogenesis and cancer, represent a precious glimmer of hope for drug discovery. Bioprospecting of organisms inhabiting the polar environments has already led to the discovery of new bioactive molecules, mainly enzymes with potential commercial use for food, paper and textile industries [4]. It is thus expected that, in the near future, natural products from polar latitudes with an untapped biotechnological potential will also be included in health products to address upcoming epidemics, and disorders related to emerging and resistant infective vectors.

## Figures and Tables

**Table 1:** Bioactive compounds from Polar marine bacteria, fungi, cnidaria, bryozoa, mollusca, echinodermata, sponges, tunicates and macroalgae.

Species	Collection Site (Distribution)	Compound	Molecule Type	Bioactivity	Reference
**BACTERIA**					
**Plylum Actinobacteria**					
**Class Actinobacteria**					
*Arthrobacter* sp.	Marine sediment, Terranova Bay, Ross Sea (Antarctica);	monoramnholipids	rhamnolipid	Antimicrobial activity against *Burkholderia cepacia* complex	[70]
	Surface water, sea ice, zooplankton, the deep sea, and meltwater (Arctic Ocean)	arthrobacilins A–C	cyclic glycolipids	Antimicrobial activity against *Vibrio anguillarum* and *Staphylococcus aureus*	[71]
*Nocardia dassonvillei*	Marine sediment (Arctic Ocean)	N-(2-hydroxyphenyl)-2-phenazinamine, 1,6-dihydroxyl-phenazine, 6-hydroxyl phenazine-1-carboxylic acid, 6-methoxy-1-phenazinol, 2-amino-1, 4-naphthoquinone, 2-amino-phenoxazin-3-one and 2-(N-methylamino)-3-phenoxazone	phenazine, phenoxazine, naphthoquinone	Antifungal activity against *Candida albicans* and high cancer cell cytotoxicity against HepG2, A549, HCT-116 and COC1 cells	[72]
*Nocardiopsis* SCSIO KS107	Seashore sediment sample, China Great Wall station (Antarctica)	7-hydroxymucidone, 4-hydroxymucidone, germicidin H	α-pyrones	Antibacterial activity against *Micrococcus luteus* and *Bacillus subtilis*	[73]
*Rhodococcus* sp. B7740	Deep seawater (Arctic Ocean)	isorenieratene (**116**)	carotenoid	Antioxidant activity	[74,75]
		menaquinone MK_8_(H_2_) (**115**)	isoprenoid quinone	Antioxidant and antiglycation activities	[76]
*Streptomyces* sp.	Marine surface sediment of the East Siberian continental margin (Arctic Ocean)	arcticoside, C-1027 chromophore-V, III, fijiolides A and B	benzoxazines, glycosylated paracyclophane	Cytotoxic activity against breast carcinoma MDA-MB231 cells and colorectal carcinoma cells (line HCT-116)	[77]
*Streptomyces* strain 1010	Shallow sea sediment from the region of Livingston Island (Antarctica)	phthalic acid diethyl ester, 1,3-bis(3-phenoxyphenoxy)benzene, exanedioic acid dioctyl ester, 2-amino-9,13-dimethyl heptadecanoic acid	aromatic compounds	No activity tested *	[78]
*Streptomyces* sp. SCO736	Marine sediment (Antarctica)	antartin (**10**)	zizaane-type sesquiterpene	Cytotoxic activity against A549, H1299 and U87 cancer cell lines by causing cell cycle arrest at the G1 phase	[79]
*Streptomyces* sp. NPS008187	Alaskan marine sediment (Arctic Ocean)	glyciapyrroles A (**11**), B (**12**) and C (**13**), cyclo(leucyl-prolyl), cyclo(isoleucyl-prolyl), cyclo(phenylalanyl-prolyl)	pyrrolosesquiterpenes, diketopiperazines	Cytotoxic activity against colorectal adenocarcinoma HT-29 and melanoma B16-F10	[80]
**Plylum Proteobacteria**					
**Class γ-Proteobacteria**					
*Pseudoalteromonas haloplanktis* TAC125	French Antarctic station Dumont d’Urville, Terre Adélie (Antarctica)	methylamine	Volatile Organic Compounds (VOCs)	Antimicrobial activity against *B. cepacia* complex	[81]
		4-hydroxybenzoic acid	benzoic acid derivative	Antitumor activity against human A459 lung adenocarcinoma cells	[82]
		cyclo-(D-pipecolinyl-L-isoleucine), cyclo-(L-prolyl-L-histidine), cyclo-(L-prolyl-L-alanine), cyclo-(L-prolyl-L-tyrosine), cyclo-(L-prolyl-L-proline), cyclo-(L-alanyl-L-isoleucine), cyclo-(D-pipecolinyl-L-leucine), cyclo-(L-pipecolinyl-L-phenylalanine), L-valyl-L-leucyl-Lprolyl-L-valyl-L-prolyl-L-glutamine and L-tyrosyl-L-valyl-L-prolyl-L-leucine	diketopiperazines	Antioxidant activity	[83]
		pentadecanal	long-chain fatty aldehyde	Anti-biofilm activity against *Staphylococcus epidermidis*	[84]
*Pseudomonas* sp.	Terranova Bay, Ross Sea (Antarctica)	monoramnholipids	rhamnolipid	Antimicrobial activity against *B. cepacia* complex	[70]
*Psychrobacter*	Marine sediment from Terranova Bay, Ross Sea (Antarctica)	monoramnholipids	rhamnolipid	Antimicrobial activity against *B. cepacia* complex	[70]
**Plylum Bacteroidetes**					
**Class Flavobacteriia**					
*Aequorivita*	Marine sediments from Edmonson Point (Antarctica)	R-(+)-N-[15-methyl-3-(12-methyltridecanoyloxy)-hexadecanoyl]glycine and methyl ester derivatives;	aminolipids	Antimicrobial activity against *S. aureus*	[85]
*Salegentibacter* strain T436	Bottom section of a sea ice floe (Arctic Ocean)	4-Hydroxy-3-nitrobenzoic acid, 4,6-Dinitroguiacol, 4,5-Dinitro-3-methoxyphenol, (4-Hydroxy-3-nitrophenyl)-acetic acid methyl ester, (4-Hydroxy-3,5-dinitrophenyl)-acetic acid methyl ester, (4-Hydroxy-3-nitrophenyl)-acetic acid, (4-Hydroxy-3,5-dinitrophenyl)-acetic acid, (4-Hydroxy-3,5-dinitrophenyl)-propionic acid methyl ester, (4-Hydroxy-3-nitrophenyl)-propionic acid, (4-Hydroxy-3,5-dinitrophenyl)-propionic acid, 2-Chloro-3-(4-hydroxy-3,5-dinitrophenyl)-propionic acid methyl ester, 2-Hydroxy-3-(4-hydroxy-3-nitrophenyl)-propionic acid methyl ester, 2-(4-Hydroxy-3-nitrophenyl)-ethanol, 2-(4-Hydroxy-3,5-dinitrophenyl)-ethyl chloride, 2-(4-Hydroxy-3,5-dinitrophenyl)-ethanol, 2-Nitro-4-(2 -nitroethenyl)-phenol, 3,5-Dinitrogenistein, 3-Nitrogenistein, 3-Nitro-1H-indole	aromatic nitro compounds	Antimicrobial activity against *C. albicans*, *Paecilomyces variotii*, *Penicillium notatumb*, *Mucor miehei* Tü 284, *Magnaporthe grisea*, *Nematospora coryli*, *Ustilago nuda*, *Bacillus brevis*, *B. subtilis*, *M. luteus*, *Escherichia coli* K12b, *Proteus vulgaris* and cytotoxic activities	[86,87]
**Plylum Firmicutes**					
**Class Bacilli**					
*Bacillus* sp.	Sea mud (Arctic Ocean)	mixirins A, B and C	cyclopeptides	Cytotoxic activity against human colon tumor cells (HCT-116)	[88]
**FUNGI**					
**Plylum Ascomycota**					
**Class Eurotiomycetes**					
*Aspergillus protuberus* MUT 3638	Sub-sea sediments, Barents Sea (Arctic Ocean)	bisvertinolone	sorbicillonoid	Antimicrobial activity against *S. aureus*	[89]
*Penicillium* sp. PR19N-1	Deep-sea sediment, Prydz Bay (Antarctica)	chlorinated eremophilane sesquiterpenes (**14–17**), eremofortine C (**18**)	chloro-eremophilane sesquiterpenes	Cytotoxic activity against HL-60 and A549 cancer cell lines	[90]
		eremophilane-type sesquiterpenes (**19–23, 25, 26**), eremophilane-type lactam (**24, 27**)	eremophilane-type sesquiterpenes	Cytotoxic activity against HL-60 and A549 cancer cell lines	[91]
*Penicillium granulatum* MCCC 3A00475	Deep-sea sediment, Prydz Bay (Antarctica)	spirograterpene A (**37**), conidiogenone I (**38**) and conidiogenone C (**39**)	tetracyclic spiro-diterpene, cyclopiane diterpenes	Spirograterpene A: antiallergic effect on immunoglobulin E (IgE)-mediated rat mast RBL-2H3 cells	[92]
*Penicillium* sp. S-1-18	Sea-bed sediments (Antarctica)	butanolide, guignarderemophilane F, xylarenone A (**28**)	furanone derivative, sesquiterpene	Butanolide: inhibitory activity of butanolide against tyrosine phosphatase 1B; xylarenone A: antitumor activity against HeLa and HepG2 cells and growth-inhibitory effects against pathogenic microbes	[93,94]
*Penicillium crustosum* PRB-2	Deep-sea sediment, Prydz Bay (Antarctica)	penilactones A and B	oxygenated polyketides	Cytotoxic activity against HCT-8, Bel-7402, BGC-823, A549 and A2780 tumor cell lines	[95]
**Class Dothideomycetes**					
Strain KF970 (Lindgomycetaceae family)	Sea-water (Arctic Ocean)	lindgomycin, ascosetin	polyketides	Antimicrobial activity against methicillin-resistant *S. aureus*	[96]
**ALGAE-ASSOCIATED MICROBES**					
**Bacteria-Plylum Actinobacteria-Class Actinobacteria**					
*Nocardiopsis* sp. 03N67	Arctic seaweed (*Undaria pinnatifida*)	cyclo-(L-Pro-L-Met)	diketopiperazine	Anti-angiogenesis activity against human umbilical vein endothelial cells (HUVECs)	[97]
**SPONGE-ASSOCIATED MICROBES**					
**Bacteria-Plylum Bacteroidetes-Class Flavobacteriia**					
*Cellulophaga fucicola*	Antarctic sea sponge	zeaxanthin (**110**), β-cryptoxanthin (**111**), β-carotene (**112**)	carotenoids	Antioxidant activity	[98]
*Zobellia laminarie*	Antarctic sea sponge	zeaxanthin (**110**), β-cryptoxanthin (**111**), β-carotene (**112**), phytoene (**113**)	carotenoids	Anti-UV and antioxidant activity and phototoxicity profile in murine fibroblasts	[99]
**Bacteria-Plylum Proteobacteria-Class γ-Proteobacteria**					
*Pseudomonas aeruginosa*	*Isodictya setifera*, Ross Island (Antarctica)	cyclo-(L-Pro-L-Val)/cyclo-(L-Pro-L-Leu)/cyclo-(L-Pro-L-Ile)/cyclo-(L-Pro-L-Phe)/cyclo-(L-Pro-L-Tyr)/cyclo-(L-Pro-L-Met)/diketopiperazines, phenazine-1-carboxylic acid, phenazine-1-carboxamide	diketopiperazines, phenazine alkaloids	Antimicrobial activity against *B. subtilis*, *S. aureus* and *M. luteus*	[65]
**Fungi-Plylum Ascomycota-Class Leotiomycetes**					
*Pseudogymnoascus* sp. F09-T18-1	Antarctic sponge genus *Hymeniacidon*, Fildes Bay, King George Island (Antarctica)	pseudogymnoascin A, B, C, 3-nitroasterric acid	nitroasterric acid derivatives	Inactive in antimicrobial activity against *P. aeruginosa*, *Acinetobacter baumannii*, *E. coli*, *S. aureus*, a methicillin-sensitive *S. aureus* and methicillin-resistant *S. aureus, C. albicans*, *Aspergillus fumigatu*	[100]
**CORAL-ASSOCIATED MICROBES**					
**Fungi-Plylum Ascomycota-Class Eurotiomycetes**					
*Penicillium* sp. SF-5995	Unidentified soft coral, Terra Nova Bay (Antarctica)	methylpenicinoline	pyrrolyl 4-quinoline alkaloid	Anti-inflammatory effect inhibiting NF-κB and MAPK pathways in lipopolysaccharide-induced RAW264.7 macrophages and BV2 microglia	[101]
**CNIDARIA**					
**Plylum Cnidaria-Class Anthozoa**					
*Alcyonium antarcticum*	Terra Nova Bay (Antarctica)	bulgarane sesquiterpene	sesquiterpene	No bioactivity tested (antipredation activity and ichthyotoxicity) *	[102]
*Alcyonium antarcticum*	Weddell Sea (Antarctica)	alcyopterosins	illudalane sesquiterpenoids	No bioactivity tested (antipredation activity) *	[103]
*Alcyonium paessleri*	South Georgia Islands (Antarctica)	alcyopterosin A (**29**), C (**30**), E (**31**), H (**32**)	illudalane sesquiterpenoids	Cytotoxic activity against Hep-2 (human larynx carcinoma) and HT-29 (human colon carcinoma) cell lines	[104]
*Alcyonium paessleri*	South Georgia Islands (Antarctica)	paesslerins A (**33**), B (**34**)	esquiterpenoids	Cytotoxic activity against human tumor cell lines	[105]
*Anthomastus bathyproctus*	Deception Island, South Shetland Islands (Antarctica)	conjugated cholestane, ergostane and 24-norcholestane steroids (**79–82**)	steroids	Cytotoxic activity against three human tumor cell lines.	[106]
*Anthoptilum grandiflorum*	Burdwood Bank, Scotia Arc (Antarctica)	bathyptilone A (**48**), B, C, enbepeanone A	briarane diterpenes, trinorditerpene	Bathyptilone A: cytotoxic activity against the neurogenic mammalian cell line Ntera-2	[107]
*Dasystenella acanthina*	Kapp Norvegia, Eastern Weddell Sea (Antarctica)	7 polyoxygenated steroids (**83–89**)	steroids	Growth inhibition of several human tumor cell lines LN-caP and K-562	[108]
*Gersemia fruticosa*	White Sea (circumpolar Arctic)	6 polyoxygenated sterols (**90–95**)	sterols	Cytotoxic activity against human erythroleukemia K-562 cells, HL-60 and P388	[109]
*Gersemia fruticosa*	White Sea (circumpolar Arctic)	9,11-secosterol (**96**)	sterol	Cytotoxic activity against human leukemia K562, cervical cancer HeLa and Ehrlich ascites tumor cells	[110]
*Gersemia fruticosa*	Alaskan Beaufort Sea (Arctic Ocean)	gersemiols A–C, eunicellol A (**40**)	diterpenoids	Eunicellol A: antimicrobial activity against MRSA—methicillin resistant *S. aureus*	[111]
*Plumarella delicatissima*	Plateau of Fascination, Falkland Islands (Antarctica)	keikipukalides A–E (**41–45**), pukalide aldehyde (**46**), norditerpenoid ineleganolide (**47**)	diterpenes, diterpenoid	Cytotoxic activity against leishmaniasis causing a parasite, *Leishmania donovani*, with no cytotoxicity against the mammalian host	[112]
Undescribed soft coral	Scotia Arc (Antarctica)	shagenes A (**35**), B (**36**)	sesquiterpenoids	Cytotoxic activity against leishmaniasis causing a parasite, *L. donovani*, with no cytotoxicity against the mammalian host	[113]
**BRYOZOA**					
**Plylum Bryozoa-Class Gymnolaemata**					
*Tegella* cf. *spitzbergensis*	Bear Island (Arctic Ocean)	ent-eusynstyelamide B, eusynstyelamides D–F	brominated tryptophan-derived	Antimicrobial activity against bacteria; weak cytotoxicity against the human melanoma A2058 cell line	[114]
*Dendrobeania murrayana*	Vesterålsfjorden, Northern Norway (Arctic Ocean)	dendrobeaniamine A	guanidine alkaloid	Tested but inactive for cytotoxic, antimicrobial, anti-inflammatory or antioxidant activities	[115]
*Alcyonidium gelatinosum*	Hopenbanken, Svalbard (Arctic Ocean)	ponasterone A and F	ecdysteroids	Tested but inactive for cytotoxic, antimicrobial, estrogen receptor agonist activities	[52]
**MOLLUSCA**					
**Plylum Mollusca-Class Gasteropoda**					
*Austrodoris kerguelenensis*	Anvers Island (Circumpolar Antarctica)	palmadorin A (**49**), B (**50**), D (**51**), M (**52**), N (**53**), O (**54**)	diterpenoid glyceride esters	Inhibition of human erythroleukemia (HEL) cells; Palmadorin M inhibits Jak2, STAT5, and Erk1/2 activation in HEL cells	[116]
**ECHINODERMATA**					
**Plylum Echinodermata-**					
**Class Holothuroidea**					
*Kolga hyalina*	Deep sea, Amundsen Basin (Arctic Ocean)	holothurinoside B, kolgaosides A (**120**), B (**121**)	triterpene holostane nonsulfated pentaosides	Kolgaosides A–B: hemolytic activity against mouse erythrocytes and inhibition against Ehrlich ascite carcinoma cells	[117]
*Staurocucumis liouvillei*	South Georgia Islands (Antarctica)	liouvillosides A (**118**), B (**119**)	trisulfated triterpene glycosides	Activity against herpes simplex virus type 1 (HSV-1)	[118]
**Class Astheroidea**					
*Asterias microdiscus*	Chukchi Sea (Arctic Ocean)	polyhydroxylated steroids A–F	steroids	No activity tested *	[119]
**Class Ophiuroidea**					
*Ophiosparte gigas*	Ross Sea (Antarctica)	cholest-5-ene-2α,3α,4β,21-tetrao1-3,21-disulphate (**102**), cholest-5-ene-2β,3α, 21-triol-2,21-disulphate (**103**)	disulfated polyhydroxysteroids	cholest-5-ene-2α,3α,4β,21-tetrao1-3,21-disulphate: cytotoxic activity; cholest-5-ene-2β,3α, 21-triol-2,21-disulphate: cytoprotective activity against HIV-1	[120]
*Astrotoma agassizii*	Antarctica	disulfated polyhydroxysteroids (**104–106**)	disulfated polyhydroxysteroids	Activity against one DNA (HSV-2) and two RNA (PV-3, JV) viruses	[121]
**SPONGES**					
**Plylum Porifera**					
**Class Demospongiae**					
*Crella* sp.	Norsel Point, Amsler Island (Antarctica)	norselic acid A (**97**), B (**98**), C (**99**), D (**100**), E (**101**)	oxidized steroids	Norselic acid A: activity against MRSA, methicillin-sensitive *S. aureus* (MSSA) and vancomycin-resistant *Enterococci faecium* (VREF) and *C. albicans*. All norselic acids were active against leishmaniasis	[122]
*Dendrilla antarctica*	Anvers Island (Antarctica)	aplysulphurin (**55**), membranoid A (**56**), B (**57**), C (**58**), D (**59**), E (**60**), G (**61**),H (**62**)	oxidized diterpenoids	Aplysulphurin: activity against *C. albicans*, and Gram-negative antibiotic activity against *S. aureus* and *E. coli*; membranoids: activity against the leishmaniasis	[123,124]
		darwinolide (**63**), tetrahydroaplysulphurin-1 (**65**), membranolide (**66**), glaciolides (**67–68**), cadlinolide C (**69**), dendrillin A (**70**), B (**71**), C (**72**), D (**73**) and semisynthetic derivatives (**74–76**)	oxidized diterpenoids	Darwinolide: selectivity against the biofilm phase of MRSA compared to the planktonic phase; membranolide: activity against MRSA; dendrillin B: activity against *L. donovani*; 76, activity against *Plasmodium falciparum*	[125,126]
		9-11-dihydrogracilin A (**64**)	oxidized diterpenoid	Immuno-modulatory and anti-inflammatory activity in human cell lines	[127]
*Geodia baretti*	North Sea off the coast of Sweden and the northern coast of Iceland (Arctic Ocean)	barettin and the geobarrettins	diketopiperazine (likely produced by a symbiont)	Moderate antioxidant and anti-inflammatory activities	[53]
					[128]
					[129]
*Haliclona viscosa*	Svalbard Archipelago (Arctic Ocean)	viscosamine	3-alkyl pyridinium alkaloids	Antibiotic activity against four separate sympatric bacterial strains	[130]
		viscosaline			[131]
*Kirkpatrickia variolosa*	Antarctica	variolins A-D (B most active)	pyridopyrrolopyrimidine	Cytotoxic activity against P388 murine leukemia cell line	[132]
					[133]
*Latrunculia* sp.	Aleutian Islands, Alaska (also found in Antarctic specimens)	discorhabdins A, C, R, dihydrodiscorhabdin B	spirocyclic imino-quinones	Anti-HCV (Hepatitis C virus) activity, antimalarial activity and selective antimicrobial activity against MRSA, *Mycobacterium intracellulare* and *M. tuberculosis*.	[134]
				Antiprotozoal activity in vitro (*P. falciparum*)	
	Weddell Sea (Antarctica)	tsitsikammamines	pyrroloiminoquinones	Anticancer and cytotoxic activities	[57]
*Lyssodendoryx flabellata*	Terra Nova Bay (Antarctica)	terpioside	glycosphingolipid	Inhibition effect in mixed lymphocyte reactions on human cells	[135]
*Mycale acerata*	Terra Nova Bay (Antarctica)	mycalol	alkyl glyceryl ether lipid	Activity against human thyroid carcinoma cells	[136]
*Plakortis simplex*	Sula Ridge Reef, Norwegian Shelf (sub Arctic)	methyl 2-((3R,6S)-4,6-diethyl-6-hexyl-3,6-dihydro-1,2-dioxin- 3-yl)acetate	cyclic peroxide (fatty acids)	Selectively inhibited proliferation in gastric cancer (GXF 251L), non-small cell lung cancer (LXFL 529L) and melanoma (MEXF 462NL) cell lines.	[137]
*Polymastia boletiformis*	Norwegian coast, Western Irish Coast (sub Arctic)	polymastiamide A (**117**), B, C, D, E, F	sulfated steroid-amino acid conjugates	Polymastiamide A: antifungal activity against plant pathogens *Cladosporium cucumerinum* and *Pythium ultimum* and human yeast pathogen *C. albicans*	[138]
				Polymastiamide A: antibacterial activity against *S. aureus*	[139]
					[140]
*Stryphus fortis*	Spitsbergen, Svalbard, (Arctic Ocean)	ianthelline	bromotyrosine derivative	Antitumor properties against several malignant cell lines and inhibition of PK activity	[141]
*Suberites* sp.	King George Isalnd, McMurdo Sound (Antarctica)	suberitenones A (**77**), B (**78**)	oxidized sesterterpenoids	Inhibition of the cholesteryl ester transfer protein (CETP)	[142]
**TUNICATES**					
**Plylum Chordata**					
**Class Ascidiacea**					
*Aplidium meridianum*	South Georgia Islands (Antarctica)	meridianins	brominated 3-(2-aminopyrimidine)indoles	Prevention of cell proliferation and induction of cell apoptosis. Inhibition of CDKs, GSK-3, PKA and other kinases in the low micromolar range	[143]
*Aplidium* sp.	Ross Sea (Antarctica)	rossinones A (**122**), B (**123**)	meroterpenoids	Antiproliferative activity against several cell lines. Antiviral activity against the DNA virus HSV-1 as well as antibacterial and antifungal activity against *B. subtilis* and *Trichophyton mentagrophytes*	[144]
*Clavelina lepadiformis*	Bergen, Norway	lepadins	decahydroquinoline alkaloid	Lepadin A: anti-cancer activity against leukemia P388, breast cancer (MCF7), glioblastoma/astrocytoma (U373), ovarian (HEY), colon (LoVo) and lung (A549)	[145]
					[146]
*Synoicum adareanum*	Anvers Island (Antarctica)	palmerolides A–G	enamide-bearing macrolides	Palmerolide A: Activity against melanoma (UACC-62 LC50), by inhibition of vacuolar ATPase-	[147]
					[148]
		hyousterones A (**107**), B, C (**108**), D, abeohyousterone (**109**)	ecdysteroids	Activity against colon cancer cells	[149]
*Synoicum pulmonaria*	Tromso, Northern Norway (Arctic Ocean)	synoxazolidinones A-C	brominated guanidinium oxazolidinones	Antibacterial activity against MSSA, MRSA and *Corynebacterium glutamicum* as well as antifungal properties against *Saccharomyces cerevisiae*. Active against human melanoma (A2058), breast adenocarcinoma (MCF-7) and colon carcinoma (HT-29) cell line, with noted cytoxicity	[150]
					[151]
		pulmonarins A, B	brominated methoxybenzoylesters bearing quaternary ammonium mioeties	Acetylcholinesterase inhibitory activity and weak antibacterial activity against *C. glutamicum*	[152]
**MACROALGAE**					
**Plylum Rhodophyta**					
**Class Florideophyceae**					
*Delisea fimbriata* and *pulchra*	Anvers Island (Antarctica)	fimbrolides and analogues	polyhalogenated furanones	Antimicrobial activity against *S. aureus*, *E. coli*, *C. albicans* and *Streptococcus* sp.	[153]
*Plocamium cartilagineum*	Anvers Island (Antarctica)	oregonene (**1**) and similar compounds (**2–4**), anverenes A (**5**), B (**6**), C (**7**), D (**8**), E (**9**)	polyhalogenated monoterpenes	Cytotoxic activity against cervical cancer cells	[154]
Plylum Ochrophyta					
Class Phaeophyceae					
*Desmarestia menziesii*	Anvers Island (Antarctica)	Menzoquinone (**124**)	terpenoid-quinone	Antimicrobial activity against MRSA, MSSA, VREF	[155]

* Natural compounds with no bioactivity tested.
